# Punishing liars—How monitoring affects honesty and trust

**DOI:** 10.1371/journal.pone.0205420

**Published:** 2018-10-10

**Authors:** Sascha Behnk, Iván Barreda-Tarrazona, Aurora García-Gallego

**Affiliations:** 1 Department of Banking and Finance, University of Zurich, Zurich, Switzerland; 2 LEE and Department of Economics, Universitat Jaume I, Castellón, Spain; Universidad de Alicante, ITALY

## Abstract

Previous experiments have shown that the possibility to punish liars does not per se increase honesty in principal-agent relationships. In this study, we first establish a punishment mechanism that substantially enhances honest behavior and trust in a sender-receiver game: the possibility to impose severe sanctions that are cost-free for enforcers. Adopting this effective mechanism, we investigate how variations in the probability of detecting lies affect sender and receiver. We find that high honesty levels persist under such punishment mechanism even when the detection probability is significantly reduced. Furthermore, the relationship between monitoring and honesty does not follow a linear trend, as a moderate monitoring level proves to be less effective in enhancing honesty than high or very low levels. The punishment mechanism has an even more robust effect on receivers, showing similarly high levels of trust independently of the detection probability. Our analysis of subjects’ beliefs provides further insights into the mechanics behind these behavioral patterns.

## Introduction

The possibility of being punished is a strong incentive to comply with social norms in human societies. It has been shown that those institutions in which punishment mechanisms are used to enhance norm compliance are widely preferred to sanction-free settings [1]. How strong the human appetite for penalizing defectors is can be best represented by the fact that people are willing to incur substantial costs to punish those who break a norm. This includes cases in which enforcers do not receive a material gain from punishing others [2–5] and cases in which they act as third parties who are not exposed to the negative consequences of the norm defection [6,7].

Despite its important role, there is also evidence that punishment is not a one-size-fits-all measure to foster pro-social actions. The behavioral consequences of potential punishments as well as the willingness to actually enforce sanctions are not only moderated by experienced emotions [8–10]. The effectiveness of punishment also seems to depend on the underlying social dilemma [11], which cannot only be attributed to different parameters of the punishment mechanism itself [12]. For instance, while punishment is an effective measure to enhance cooperation in public goods settings even in the long-run [13], it reduces pro-social behavior in other settings such as the trust game [14]. A similar issue is observable with respect to deception. Overall, there is only a limited number of studies investigating the effects of potential punishment on deception and many of them show that punishment does not lead per se to diminished lying (see, for instance, [15,16]).

We contribute to the literature by conducting an economic experiment in which we investigate the effect of sanctions on deception in two ways. First, we test whether an efficient punishment mechanism enhances honesty and trust in principal-agent relationships: the possibility to impose severe sanctions on dishonest agents that are cost-free for the enforcing principals. Second, adopting this effective mechanism, we address a factor of fundamental importance for the functioning of a sanction system in principal-agent relationships: monitoring. Specifically, we test how robust the deterrence effect of the punishment mechanism is when we gradually reduce the probability of agents getting caught lying.

Specifically, we use a sender-receiver game based on [17] that follows [18] in additional design features. In this two-player one-shot game, there are three options with different payoffs. An agent (the sender) is informed about these payoffs while his principal (the receiver) is not. One option provides the highest payoff to the sender, one provides the highest payoff to the receiver, and a third option is Pareto-dominated. The only information the receiver gets with regards to these payoffs is a message transmitted by the sender, in which he can either honestly recommend the most favorable option for the receiver or lie by recommending one of the other two options, including the most favorable one for himself. The receiver then decides upon accepting or rejecting the message, which determines the payoffs for both parties.

While punishment is not possible in our baseline treatment, we further employ a treatment in which receivers always have the cost-free possibility to severely sanction dishonesty by reducing the sender’s final payoff to an amount that is lower than all other payoffs in the game. The comparison of these two treatments serves to generally test the severe and cost-free punishment mechanism that we propose. Previous literature indicates that such a strong sanctioning system is not only crucial for the general credibility of a punishment threat [19], but also that less strong sanction systems may not have a noticeable effect on deception and trust at all. In contrast to previous studies, in our design the ex post disclosure of sender behavior, necessary for the enforcement of sanctions, is also present in the baseline without punishments. Hence, this setting enables us to disentangle the pure deterrence effect of punishment from the image concerns caused by the ex post disclosure [18].

With regards to the second aim of this study, we note that in some settings of cooperation it is possible to eventually detect defectors without bearing significant monitoring costs, as for instance in the case of free-riding in public goods. However, deception is inevitably linked to an exploitation of asymmetric information and, hence, difficult to detect in the direct aftermath of an economic interaction. Famous examples are the market for lemons [20] or the selling of sub-optimal products in the financial services sector [21]. Perfect monitoring in principal-agent relationships can, therefore, be costly [22,23], if not impossible. The question that arises is whether the threat of a strong and cost-free punishment mechanism is sufficient to keep agents from deceiving their principals even if their honesty or dishonesty is not disclosed in each and every case. This is particularly important regarding the existence of thresholds above which monitoring is able to create crowding-out effects, in the sense that agents can even reduce their norm adherence when they are monitored in excess [24,25].

To investigate the extent to which monitoring can be reduced without jeopardizing high honesty levels in the presence of our efficient punishment mechanism we employ three treatments—in addition to the above-mentioned treatment with punishment and 100% detection probability—in which we gradually reduce the probability with which dishonest senders are revealed to receivers to 50%, 25%, and 10%, respectively. A comparison among the four punishment treatments enables us to investigate the effect of decreased monitoring on the deterrence of the proposed punishment system.

Furthermore, [26] have shown that the size of a lie matters for receivers in the sense that senders were punished harder the more they tried to gain from a lie. We address this issue from the sender's point of view regarding two types of lies: a lie that leads to profitable deception and a lie that promotes an equalization of payoffs on a Pareto-dominated level.

Moreover, we use the strategy method in each treatment by letting senders make a decision on which message should be sent to the receiver in three different payoff scenarios. While each scenario includes the three above-mentioned options, we vary the total sum of payoffs and the difference between the payoffs that both players can gain from the options. These scenarios are similar to the ones used in [17]. However, while [17] employed each scenario in a separate treatment, we use a within-subject setting. After the sender made a decision in each scenario, one scenario was randomly selected and the respectively chosen message was sent to the receiver. The application of a punishment mechanism to different payoff scenarios enables us to examine in detail how various monetary temptations and different consequences for their counterparts influence the senders' behavior when punishment of any lie is possible.

For the case of assured revelation of dishonesty, we find that senders choose honest messages substantially more often when punishment is possible. This result confirms that, in contrast to many other punishment calibrations in the literature, our highly efficient sanction system provides a credible threat to potentially deceptive agents.

With respect to the variation in detection probabilities, we observe similarly high fractions of honest messages comparing assured revelation with 50% detection probability. Strikingly, the high level of senders’ honesty is still maintained in most payoff scenarios when the detection probability is reduced to only 10%. However, our results also show that the relationship between monitoring and honesty does not follow a linear trend as moderate monitoring proves to be less effective in enhancing honesty than high or low monitoring levels. The treatment with 25% detection probability shows rates of honesty that are significantly lower than in the case of assured revelation.

After senders made their decisions but before informing them of the payoff scenario that was randomly implemented and, hence, the message that was finally sent to the receiver, we elicited their beliefs regarding their counterpart’s behavior and expectations as well as regarding the behavior of other senders. Our analysis of these beliefs rules out that the stable deterrence effect of punishment is due to changes in their strategic considerations, leaving senders’ anticipated psychological costs of being punished for deception as a potential explanation and, thus, probably turning the decision whether to be honest or not into a deterministic one. Also overweighting of small probabilities (see, for instance, [27]), in line with prospect theory [28], could in some way account for the fact that we observe a high deterrence effect with very low detection probability (10%).

These findings suggest that monitoring and the related costs in principal-agent relationships could be considerably reduced without risking a major decline in honesty under a severe and, for the enforcer, cost-free punishment mechanism.

Moreover, our within-subject comparison of different payoff scenarios reveals that the deterrence effect of punishment is always significant but less strong when senders are able to gain a comparatively high amount from deception. On the other hand, differences in the financial consequences for the receiver do not significantly affect sender decisions in our punishment setting. We also find that the second alternative to honesty, sending payoff-equalizing messages, even if frequently chosen in the baseline without sanctions, nearly disappears with the possibility of punishment. Our analysis of individual beliefs reveals that punishment works as a positive selection screen in this regard since it eliminates strategic sender actions in terms of falsely promoting an equal outcome in order to maximize profits.

In line with sender behavior, receivers show substantially higher trust levels when severe and cost-free punishment is possible. Furthermore, we do not find a significant difference in message acceptance rates between any of the punishment treatments, indicating that receivers expect a similarly strong deterrence effect of punishment on sender behavior, independently of the detection probability. This is an important finding since many aspects of economic cooperation are built on trust and can only be established if the involved parties actually agree to interact with each other.

This paper is structured as follows: In the second section we review the related literature. The third section describes the experimental design and procedures. Hypotheses are derived from a subjective equilibrium analysis in the fourth section. In the fifth section we present and discuss our results, and finally we close with the conclusions. The experimental instructions can be found in S1 Appendix.

## Literature review

The deterrence effect of punishment is sensitive to the underlying social dilemma [11–12]. This means that a decision about whether to enforce a sanction is not only influenced by the monetary loss inflicted, but also by the way the defector intended to obtain this amount. A particular case seems to be defection that goes along with deceptive behavior. For instance, individuals are more inclined to punish defectors when their selfish act was preceded by a deceptive message [26,29].

The number of experimental studies that investigate deterrence in the deception context by comparing punishment settings with sanction-free baselines is limited, as summarized in Table 1. The first group of articles does not find a significant reduction in deception when agents face the possibility of being sanctioned for deceiving their principals. [15] used a repeated sender-receiver game with alternating roles in which both players' payoffs were reduced to zero in case the receiver punished her counterpart. Although receivers showed more trust when punishment was possible, the authors did not find a significant difference in truth-telling between their baseline and the punishment treatment.

**Table 1:** Parameters of selected experimental studies examining punishment effects on deception.

	**Sánchez-Pagés & Vorsatz (2007)**	**Sánchez-Pagés & Vorsatz (2009)**	**Peeters et al. (2013)**
**Game structure**	2 options with misaligned payoffs	2 options with misaligned payoffs	2 options with misaligned payoffs
**Ex ante transparency** (conflicts of interest)	yes	yes	yes
**Ex post transparency** (conflicts of interest)	yes	yes	yes
**Punishment severity**	100% of the earnings	100% of the earnings	100% of the earnings
**Punishment costs**	100% of the earnings	100% of the earnings	100% of the earnings
**Punishable actions**	all	all	all
**Number of rounds**	50	50	60 + 40
**Alternating roles**	yes	yes	yes
**Specific features**		costly option to remain silent	institution choice in later stages
**Findings regarding Punishment**	punishment does not reduce deception	punishment does not reduce deception	punishment does not reduce deception, except for self-selected punishment groups in the institution selection phase
	**Church & Kuang (2009)**	**Xiao (2013)**	**Kimbrough & Rubin (2013)**
**Game structure**	value assessment (coins)	2 options with misaligned payoffs	trust game with pre-play messages
**Ex ante transparency** (conflicts of interest)	no/yes	no	yes
**Ex post transparency** (conflicts of interest)	yes	enforcers: yes receivers: no	yes
**Punishment severity**	25 out of {100–140}	50% of the earnings	20% of initial endowment + outstanding amount
**Punishment costs**	10 out of {100–200}	no costs	20% of initial endowment if jury denies punishment
**Punishable actions**	dishonesty	all	dishonesty
**Number of rounds**	1	3 scenarios	10
**Alternating roles**	no	no	no
**Specific features**	outside option for receivers; punishment decision before final outcome is shown	third-party punishment; varying punishment profitability; varying receiver information	jury system
**Findings regarding Punishment**	punishment reduces deception only with ex ante disclosure of conflicts of interest	punishment reduces deception; but no effect when punishment is profitable or when receivers are not informed of punishments	punishment reduces deception from the beginning

[30] found the same pattern in a subsequent study in which senders had the possibility to remain silent as an alternative to sending a message to the receiver. [16] modified the design of [15] by randomly assigning subjects to settings with and without punishment and allowing players to choose between these institutions in later stages. Again, they found no overall difference in truth-telling with and without punishment. By analyzing subgroups of punishers and non-punishers, they showed that only in the self-selected punisher group the truth was told significantly more often when sanctioning was possible.

Furthermore, [31] investigated how deception is affected by sanctions in combination with *ex ante* disclosure of conflicting interests, which can increase deceptive behavior through "moral licensing” [32]. Receivers had to estimate an unknown value and received an advice from a better informed sender. In case a receiver punished a dishonest sender, she incurred a relatively low cost of 10% of her initial endowment and the sender's earnings were reduced to a comparably low extent, that is, by one quarter of his endowment. The possibility to get punished alone did not reduce the bias in the senders’ advice. On the other hand, the sanction system led to a significant reduction in the bias when receivers were informed about the senders’ conflict of interest from the beginning. Their explanation for this effect is that "with common knowledge of incentives, sanctions provide a real threat that regulates behavior" ([31]: p. 512).

[33] also show a deterrence effect of punishment in a deception game with cost-free third-party punishment that halved the sender's payoffs in case it was enforced. Deception rates decreased when receivers observed whether the enforcer decided to punish the sender before making their decisions. However, when enforcers were able to gain profits from punishing senders or when receivers were not aware of the punishment possibility, sanctions did not reduce deception compared to the baseline. Hence, Xiao supports the communication function of punishment regarding social norms. In a subsequent study, [34] showed that the suboptimal effect of profitable punishment can be overcome when punishers have to justify their decisions.

[35] found a positive effect of punishment in a dynamic environment. The authors used a repeated trust game with pre-play messages in order to compare the effects of costly in-group punishment depending on a jury decision and the provision of information about the other group members' behavior. In case of a positive jury decision, the deceiver had to pay the outstanding amount plus the cost of one fifth of the initial endowment that the victim had to disburse for initiating the jury process. They found that players deceived significantly less when the sanction system was applied and that both factors, punishment and information provision, worked in a mutually reinforcing way. While information sharing facilitates reputation building over time, punishment reduced deception already from the beginning, implying that its deterrence effect is not affected by repeated interactions.

In another dynamic setting, [36] showed that lying became unprofitable over time since players were inclined to report deceivers to a central authority in order to get them punished. However, when members were able to select who else to include in their group, whistle-blowers were often avoided, leading to groups in which the sanctioning mechanism lost its functionality. Punishment was also used in other contexts that include information transmission to investigate, for instance, the effect of confessions [37] and apologies [38] on individual behavior.

Overall, previous experiments led to mixed results regarding the deterrence effect of punishment in the deception setting. It is striking that, in those studies that do not show an effect of sanctions on deception, punishment either includes substantial costs for the enforcer [15,16,30] or leads to comparably slight consequences for punished senders [31]. Punishment only affected sender behavior positively in case the credibility of its threat was obviously increased, e.g. through self-selection into a punishment institution or ex-ante disclosure of conflicts of interest between agent and principal. Although the results are not directly comparable, due to different design features that might have influenced the anticipation of being punished in various ways, we can conclude that effective sanctioning of deceptive behavior is related to a strong reduction in earnings and a comparatively low cost for the enforcer. This tendency is in line with [19,39], who found that decreasing enforcement costs and, respectively, a higher severity of sanctions enhances cooperation. In order to reflect this tendency in our design, we use a punishment mechanism that reduces the final earnings of a dishonest sender considerably while, at the same time, its enforcement is cost-free for receivers.

Subsequently, we use our design to investigate how the probability with which a receiver learns about a sender's honesty or dishonesty affects deterrence. The economic analysis of how detection probabilities affect the willingness to act antisocially goes back to the seminal work of [40] about the economics of crime. The author predicts that fewer people become delinquent when the probability or the severity of punishment increases, based on economic rationality and risk attitudes. [41] compare severity and certainty of sanctions using a stealing game and obtain results that contradict Becker's predictions, except for the presence of very strong incentives. While [42] do not find a difference in the deterrence effect of the two factors, some experimental investigations confirm a relatively higher importance of detection probabilities, for instance in [43], while others show the opposite effect [44,45]. Overall, certainty and severity of punishment seem to exhibit interaction effects [46], which are most certainly moderated by various factors, such as risk attitudes [47], individual criminal history [48] as well as learning over time [49].

Importantly, while several field studies provide evidence for a positive relationship between detection probability and crime reduction, for instance regarding free-riding in public transportation [50], other studies find contradictory results in the sense that crime rates actually increase with a higher probability of detection [51]. Both earlier [52] and more recent reviews [53] of studies on the economics of crime confirm the mixed results in this regard. Given these inconclusive findings and the particular role of punishment in the deception context, indicated by previous studies, we explore the effect of severe and cost-free punishment on deception in a sender-receiver game under different probabilities of ex post disclosure in this study.

## Materials and methods

The experiment was conducted with a written consent obtained from each participant in accordance with the Declaration of Helsinki. Every potential participant in the experiments carried out at the Laboratory of Experimental Economics (LEE) at University Jaume I fills in an online form voluntarily and personally in order to be taken into consideration as experimental subject. Prior to being able to enter their data in our database, potential participants have to click on a button to confirm that they agree with the rules to participate in an economic experiment and with the privacy policy that they have just read. See webpage http://www.leerecs.uji.es/orsee/public/participant_create.php?language=en. In a second step, they are invited via email to take part in an experimental session, they have to enter the online system personally with their password, and select one of the active sessions only if they are interested. No penalties are applied if a registered subject does not want to participate in an experiment. No deception takes place in any experiment run at the LEE. The study was carried out in accordance with the recommendations of the Universitat Jaume I’s ethical committee.

### Sender-receiver game

In this one-shot game, all subjects were randomly assigned the role of either a sender or a receiver, neutrally named Player 1 and Player 2. A sender and a receiver from the same session were randomly matched. The sender was then provided with information about the payoffs that both players could get from a set of options. The receiver's task was to implement one of the options without having any information about the corresponding payoffs and by doing so she was determining both players’ payoffs. In this situation of information asymmetry, the sender transmitted a message to the receiver in which he recommended one of the options as the one that provides the highest payoff for the receiver. This message could either be honest or a lie and, since payoffs were misaligned, the sender had an economic incentive to be dishonest.

We used the strategy method and presented three different payoff scenarios to the sender. Each scenario included three options with a payoff for each player as shown in Table 2. The scenarios were based on the seminal design of [17] in combination with a Pareto-dominated third option similar to [21,54]. The dominated option was added to reduce the effect of "deception by telling the truth" [55] and to allow senders to promote an equal split of the payoffs between both players on a comparatively lower level. The three scenarios were presented on different screens and we controlled for order effects regarding their appearance as well as for the option order within a scenario. Receivers only saw the general structure of the scenarios and options without payoff information in the experiment instructions (see S1 Appendix).

**Table 2:** Sender and receiver payoffs by scenario and option (in euros).

Scenario	Option	Payoff sender	Payoff receiver
1 (low+;low-)	A	5	6
	B	6	5
	C	3	3
2 (low+;high-)	A	5	15
	B	6	5
	C	3	3
3 (high+;high-)	A	5	15
	B	15	5
	C	3	3

While the dominated option C always provided an equal payoff of three euros to both players, their interests were misaligned between options A and B and the intensity of this misalignment varied across the scenarios. In scenario 1, a successful deception led to a comparatively low additional gain of one euro for the sender compared to an equally low loss for the receiver. For this reason, we labeled this scenario (low+,low-). The label indicates how much senders earn (+) and receivers lose (-) from option B compared to option A.

In scenario 2, the sender obtained the same profit from implementing option B as in scenario 1 but now at the expense of a higher comparative loss of ten euros for his counterpart (low+,high-). Finally, in scenario 3, the sender was able to gain an additional profit of ten euros from option B, which is higher than in the other two scenarios, at the cost of his counterpart's loss which is equally high as in scenario 2 (high+,high-). In each of the payoff scenarios, the sender selected one of the following three messages to be sent to the receiver afterwards:

Message 1: Option A will earn you more money than the other two options.
Message 2: Option B will earn you more money than the other two options.
Message 3: Option C will earn you more money than the other two options.

Since each of the three options provided a different payoff to the receiver, only one of the three messages was true (henceforth called "honest message"). The remaining messages were lies that can be characterized by their payoff structure as a "deceptive message" in case the sender recommended the option that provided him with the highest payoff within a scenario and as a "payoff-equalizing" message when referring to the option that provided equal payoffs on a low level for both players.

After the sender chose a message in each payoff scenario, the computer randomly selected one of the scenarios and sent the corresponding message to the receiver, who either accepted or rejected it. In case the receiver accepted the message, the recommended option was implemented and determined the payoffs for both players. In case of rejection, one of the two non-recommended options was randomly implemented by the computer to reduce the effect of strategic truth-telling. Before informing subjects about the implemented option and presenting them their payoffs, we elicited their beliefs.

### Treatments

We conducted four treatments using the above-mentioned sender-receiver game in which we introduced the possibility for receivers to punish dishonest senders and among which we varied the probability with which receivers learned, at the end of the game, the actual sender behavior. As summarized in Table 3, the detection probabilities in these treatments were 100%, 50%, 25% and, respectively, 10%. Particularly, the receiver was always shown her own payoff on the final screen and, additionally, all payoffs of both players in each of the options in the selected scenario. In this way, in case of a revelation, she could learn whether or not the message she received from the sender was dishonest. These treatments are henceforth labeled with the letter “P” (for punishment possibility) and the respective detection probability.

**Table 3:** Treatment characteristics.

Treatments	Detection Probability (%)	Punishment Possibility	N
T100	100	no	144
P100	100	yes	60
P50	50	yes	64
P25	25	yes	60
P10	10	yes	60

While we conducted all four P-treatments exclusively for this study, we use data from one treatment of the anonymous two-player sender-receiver game with ex post disclosure of [18], which is identical to the game structure described above, but where subjects never had the possibility to punish liars. This treatment serves as our baseline without sanctions. It includes assured ex post disclosure as in P100 in the sense that the receiver always found out about the honesty or dishonesty of the sender after the game was played (henceforth called T100, for 100% ex-post disclosure).

In all treatments, both player types were informed in the experiment instructions about the potential ex post disclosure of sender behavior and the corresponding probability (see S1 Appendix).

### Punishment mechanism

In all four P-treatments, receivers were able to punish dishonest senders after the game was played by reducing the sender’s payoff to 2 euros, independently of the underlying payoff scenario. Hence, the reduction in the sender’s payoff caused by punishment would be of 4€ in scenarios 1 and 2, where the sender’s gain from lying is 6€, and of 13€ in scenario 3, where the sender’s gain from lying is 15€.

We chose the specific amount of 2 euros as the sender’s final payoff in case of a punishment for two reasons. First, to allow receivers to severely sanction senders as a condition for efficient punishment, which, according to our literature review, is necessary to successfully enhance honesty in principal-agent relationships. Second, we chose this amount since there would not have been a credible threat for sanctioning the sending of payoff-equalizing messages if punishment would have reduced the payoff to an amount equal to or higher than 3 euros.

Sanctioning senders is cost-free for receivers in order to further enhance the above-mentioned punishment efficiency. This design feature is in line with previous studies focusing on punishment in the deception domain [33,35]. Even in modern societies, efficient norm enforcement by punishment requires a certain level of effort by individuals and law enforcement institutions. What we refer to with our punishment mechanism is a system in which these costs exist, but a victim of a clear norm defection does not have to bear them individually, as it is the case in many countries. One example for such a system is when the winning party of a civil law suit does not have to bear the costs for her lawyer or the trial itself. Instead, these costs have to be covered by the losing party. A concrete example for our experimental setup in this regard is the relationship between a financial advisor and a customer, where the customer may find out that the advisor recommended suboptimal financial products in order to receive higher commissions. As a punishment, the customer could withdraw all invested money from the advisor’s institution, independently of the amount of money the customer lost due to bad counseling.

As in P100, in the P50, P25 and P10 treatments a receiver could sanction a dishonest sender when she accepted a deceptive or payoff-equalizing message, but only in case the payoff structure was actually revealed to her, which happened with the respective detection probability. No punishment was possible in case an honest message was sent or the receiver rejected the transmitted message, which is a similar procedure to those in previous studies [29]. No possibility for retaliation or reputation building exists in our design since the game was played only once.

In this aspect, our design differs from the common view in many legal frameworks, that even an attempt to harm others can be penalized. However, this rule does not apply to lies per se as a liar can only be held responsible in a legal way for causing damage in case others base their actual behavior on the lie (except for perjury). For instance, in a situation in which an advisor recommends a suboptimal financial product to an investor, rejecting this recommendation can be interpreted as a cancellation of the economic relationship between both parties. Such a situation is not in the focus of the present experiment which investigates the effect of punishment on lies that lead to a reduction in financial rewards for others. Furthermore, studies like [15,16] have shown that the willingness to punish dishonesty does not only depend on the action of the defector but also on the behavior of the victim. In their experiments, the sanction rate after the sequence lie-distrust was comparatively low, indicating that this action sequence was not worth punishing for the vast majority of their subjects.

### Belief elicitation

Given the importance of individual expectations for subjects' decisions in environments with asymmetric information, we elicit a series of subjects’ beliefs to examine their potential role as moderators of behavior in our experiment. A variety of studies have shown the importance of belief elicitation in this context, such as [56–61]. Therefore, we asked subjects to answer several questions regarding their expectations after they made their decisions but before showing them their final outcome. First of all, we asked senders if they expected the receiver with whom they were matched to accept the message they had sent (henceforth called first-order beliefs). Correspondingly, receivers were asked if they believed that the sender they were matched with had sent a truthful message.

We further elicited how much senders thought their counterpart expected to earn from the sent message in relation to the sender’s payoffs from that message, henceforth called second-order beliefs. Specifically, we asked “what do you think Player 2 expects to earn from your message?” using a five-point Likert scale with the categories “much less than I”, “less than I”, “the same as I”, “more than I” and “much more than I” as the set of possible answers. Correspondingly, we asked receivers about their relative monetary expectations “what do you expect to earn from Player 1’s message?” using a five-point Likert scale with the categories “much less than Player 1”, “less than Player 1”, “the same as Player 1”, “more than Player 1” and “much more than Player 1” as the set of possible answers.

Since not only the anticipated actions of counterparts but also of peers might have an influence on decision-making in the sense of a norm signaling, we also elicited subjects’ expectations about the behavior of other players in their role, henceforth called peer beliefs. Therefore, we presented senders again the three payoff scenarios on different screens and asked them in each screen to estimate on a four-point Likert scale from “unlikely” to “very likely” whether other senders sent a message that favored them in the respective scenario. Receivers were asked about how likely they believed it was that other receivers had accepted the message sent to them using the same answer categories as for senders.

Finally, in addition to the three belief types above, we asked senders to what extent they have considered the possibility of the receiver punishing them for deception in their message decision, using a four-point Likert scale from “not at all” to “a lot”. Using the same scale, receivers were asked to what extent they considered how much money their counterpart made them lose when deciding whether to punish a dishonest sender.

### Sample and procedures

The experiment was conducted at the LEE—Laboratory of Experimental Economics at University Jaume I, Castellón, Spain. Regarding our sample, we recruited 436 undergraduate students with the Online Recruitment System for Economic Experiments ORSEE [62]. 51.8% were female and subjects were on average 22.2 years old. Moreover, 37.1% of the subjects stemmed from the economics, management or finance fields and 34.3% stated that they were working or financed by a university fellowship. Ten sessions were run—three in the T100 treatment and two in each of the four P-treatments, each lasting about 45 minutes.

Upon arrival, subjects entered the laboratory one by one and chose a seat in front of a computer. The experiment was programmed in z-Tree [63] and the pairs as well as the player roles were randomly assigned to the seats. After the experimental instructions were read aloud, subjects answered a control question using the computers. In case subjects did not answer correctly, one of the experimenters went to their seats and clarified the question individually to ensure a full understanding of the game. After the game was played, subjects filled in a final questionnaire in which we elicited socio-demographic and further information including age, gender, major of study, whether they have a job or a university fellowship and number of siblings. At the end of each session, we paid the subjects anonymously in cash. The average earnings were around 10 euros.

## Predictions

### Senders

We derive our hypotheses regarding sender behavior from an expected utility analysis. In each scenario *i* ∊ {1,2,3}, the sender selects a message *m*_*i*_ to be sent to his counterpart, promoting one option from the set of options *Z* = {*A(honest)*, *B(deceptive)*, *C(payoff-equalizing)*} which provide monetary payoffs *π*_i_*(z)* > 0. Since senders gain the highest payoff from successful deception and both payoffs from deception and honesty are greater than the one from payoff-equalization in every scenario, we set *π*_*i*_*(B)* > *π*_*i*_*(A)* > *π*_*i*_*(C)* for the sender.

The receiver, although not provided with information about the payoffs from accepting each of the possible messages, has a say in the outcome by either accepting or rejecting the message, an element that a rational sender takes into account. Therefore, we add the sender's first order beliefs to our analysis in terms of the subjective probability *p* ∊ [0,1] with which he believes the receiver will accept the message. The sender's expected utility *EU*_*T*_ under risk neutrality in T100, our treatment without punishment possibilities, obtained from sending message *m*_*i*_ = *z* is reproduced in Eq (1).

$$EU_{T100}(m_{i}=z)=p\pi_{i}(z)+(1-p)\frac{\sum_{j\neq z}\pi_{i}(j)}{2}$$ (1)

**Fig 1:**
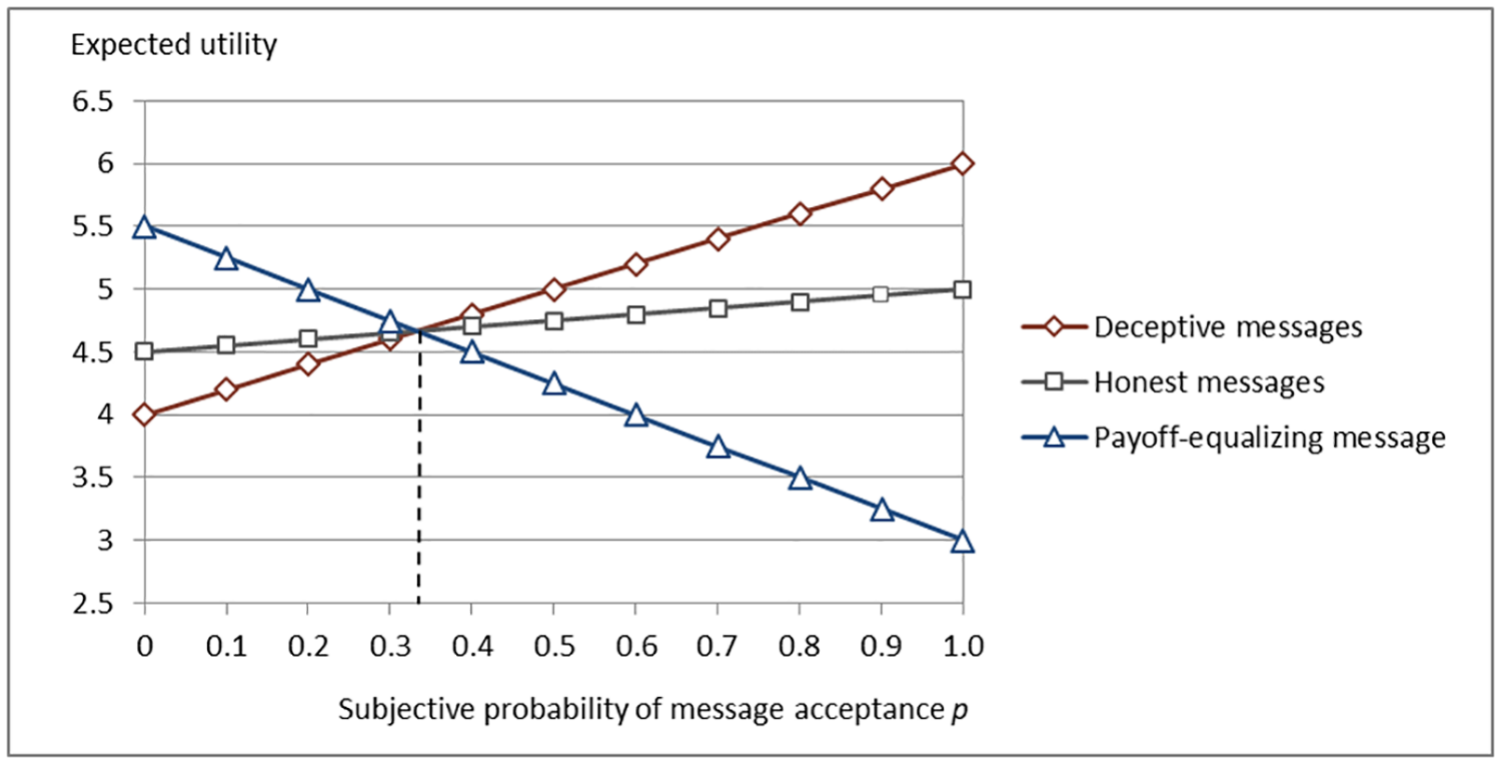
Sender's expected utility from sending messages in T100.

According to this model, a sender would never send honest messages in any scenario of the T-treatment except for the indifference point *p* = 0.33, as illustrated in Fig 1 and shown in S1 Appendix, since:

$$EU_{T100}(A)(\begin{array}{c} <EU_{T100}(B)\\ <EU_{T100}(C)\\ =EU_{T100}(B)=EU_{T100}(C) \end{array}\begin{array}{c} if\\ if\\ if \end{array}\begin{array}{c} p>0.33\\ p<0.33\\ p=0.33 \end{array}$$ (2)

In the P-treatments, the receiver has the cost-free possibility to sanction her counterpart after accepting a dishonest message by reducing his payoff from the implemented option to *π*_*S*_ < *π*_*i*_*(z)*. Since sending an honest message cannot be punished, the sender's expected utility from this action is the same as in T100. On the other hand, the expected utility from sending a deceptive or a payoff-equalizing message depends on the sender's subjective probability *q* ∊ [0,1] of the receiver punishing him after sending one of these two messages in P100, which we include in Eq (3).

$$EU_{P100}(m_{i}=z\neq A)=p(q\pi_{S}+(1-q)\pi_{i}(z))+(1-p)\frac{\sum_{j\neq z}\pi_{i}(j)}{2}$$ (3)

We show that sending honest messages becomes a dominant strategy for specific *p*-ranges in P100 by comparing the intersections of the expected utility functions between P100 and T100.

Let us define two indifference values of *p*:

*p*_*AB*_: the value of *p* at which the expected utility from truth-telling *EU*_*T*_*(A)* = *EU*_*T*_*(B*), the expected utility from sending a deceptive message.
*p*_*AC*_: the value of *p* at which *EU*_*T*_*(A*) = *EU*_*T*_*(C*), the expected utility from sending a payoff-equalizing message.

In Eq (4), we show that in T100 the difference is zero between *p*_*AC*_, up to which sending payoff-equalizing messages dominates, and *p*_*AB*_, as of which sending deceptive messages dominates, leaving no value of *p* for which honesty provides the highest expected utility. The same is true for the P-treatments when *q* = 0, i.e., in case senders do not expect to be punished for dishonesty.

$$p_{AB,T100}-p_{AC,T100}=\frac{\pi_{\bar{A}}-\pi_{\bar{B}}}{\pi_{\bar{A}}-\pi_{\bar{B}}+\pi_{i}(B)-\pi_{i}(A)}-\frac{\pi_{\bar{C}}-\pi_{\bar{A}}}{\pi_{\bar{C}}-\pi_{\bar{A}}+\pi_{i}(A)-\pi_{i}(C)}=0$$ (4)

with $\pi_{\bar{A}}=\frac{\pi_{i}(B)+\pi_{i}(C)}{2}$, $\pi_{\bar{B}}=\frac{\pi_{i}(A)+\pi_{i}(C)}{2}$, $\pi_{\bar{C}}=\frac{\pi_{i}(A)+\pi_{i}(B)}{2}$, where $\pi_{\bar{C}}>\pi_{\bar{A}}>\pi_{\bar{B}}$

However, if a sender believes that *q* > 0, positive value ranges of *p* exist that increase with *q* and within which honesty is the dominant strategy in P100. This is shown in Eqs (5) and (6), in which we compare the values of *p* of the expected utility function intersections between T100 and P100:

$$p_{AB,T100}-p_{AB,P100}=\frac{\pi_{\bar{A}}-\pi_{\bar{B}}}{\pi_{\bar{A}}-\pi_{\bar{B}}+\pi_{i}(B)-\pi_{i}(A)}-\frac{\pi_{\bar{A}}-\pi_{\bar{B}}}{\pi_{\bar{A}}-\pi_{\bar{B}}+\pi_{i}(B)-\pi_{i}(A)-q(\pi_{i}(B)-\pi_{S})}<0$$ (5)

$$p_{AC,T100}-p_{AC,P100}=\frac{\pi_{\bar{C}}-\pi_{\bar{A}}}{\pi_{\bar{C}}-\pi_{\bar{A}}+\pi_{i}(A)-\pi_{i}(C)}-\frac{\pi_{\bar{C}}-\pi_{\bar{A}}}{\pi_{\bar{C}}-\pi_{\bar{A}}+\pi_{i}(A)-\pi_{i}(C)+q(\pi_{i}(C)-\pi_{S})}>0$$ (6)

**Fig 2:**
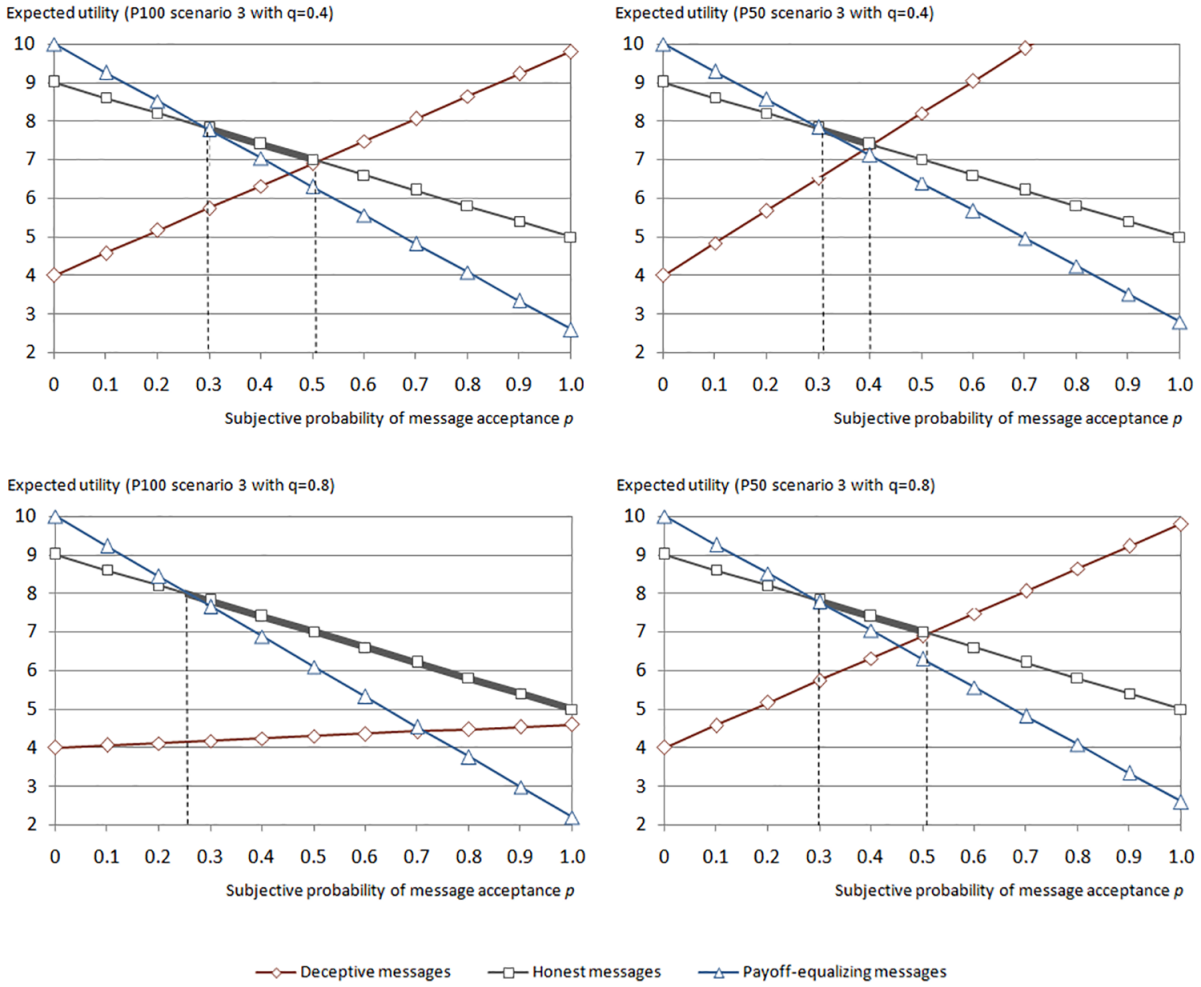
Sender’s expected utility in P100 and P50 with low (*q* = 0.4) and high (*q* = 0.8) subjective punishment probability *q* in scenario 3.

The difference *p*_*AB*,*T*_—*p*_*AB*,*P100*_ is negative since $\pi_{\bar{A}}>\pi_{\bar{B}}$ and *π*_*i*_*(B)* > *π*_*S*_, which is equivalent to a rightward shift of the intersection point in P100 compared to T100, with punishment reducing the *p*-range within which sending deceptive messages is a dominant strategy. Correspondingly, the difference *p*_*AC*,*T100*_—*p*_*AC*,*P100*_ is positive since *$\pi_{\bar{A}}<\pi_{\bar{C}}$* as well as *π*_*i*_*(C)* > *π*_*S*_ and reflects a leftward shift of the intersection point in P100 compared to T100, which leads to a comparatively smaller *p*-range within which sending payoff-equalizing messages dominates. As a consequence, truth-telling dominates between both intersection points *p*_*AC*,*P100*_ and *p*_*AB*,*P100*_ in P100. The left part of Fig 2 illustrates this pattern for two selected *q*-values in P100.

We show the pattern for representation reasons in payoff scenario 3. While equalizing messages still dominate on the left side of the *p*-distributions, sending deceptive messages can even become a completely dominated action when the expected probability of punishment *q* is sufficiently high. Therefore, we expect to find a higher level of honesty when receivers are able to punish dishonest senders compared to the baseline without a sanction system.

***H1***: *Senders will be more likely to send honest messages in P100 than in T100*.

In the following, we demonstrate that a decreasing probability of disclosing dishonesty reduces the *p*-range in which honesty is a dominant strategy in our setting by comparing the intersections of the expected utility functions between the P-treatments. Therefore, we exemplarily compare P100 and P50. In P50, the possibility to punish also depends on the 50% detection probability in the sense that a receiver can sanction a liar after acceptance of his message only if she becomes aware of his dishonesty, thanks to probabilistic disclosure, as included in Eq (7).

$$EU_{P50}(m_{i}=z\neq A)=p(0.5\pi_{i}(z)+0.5(q\pi_{S}+(1-q)\pi_{i}(z))+(1-p)\frac{\sum_{j\neq z}\pi_{i}(j)}{2}$$ (7)

We obtain that *P*_ABP,50_ < *P*_AB,P100_ for all *q* > 0 as represented in Eq (8) since *π*_*i*_*(B)* > *π*_*i*_*(A)* > *π*_*S*_ and, hence, whenever the sender expects to be punished by the receiver with a positive probability, the *p*-range in which sending a deceptive message dominates is wider in P50 than in P100 as illustrated in Fig 2.

$$\begin{array}{l} P_{AB,P50}=\frac{\pi_{\bar{A}}-\pi_{\bar{B}}}{\pi_{\bar{A}}-\pi_{\bar{B}}+\pi_{i}(B)-\pi_{i}(A)-0.5q(\pi_{i}(B)-\pi_{S})}\\ <p_{AB,P100}=\frac{\pi_{\bar{A}}-\pi_{\bar{B}}}{\pi_{\bar{A}}-\pi_{\bar{B}}+\pi_{i}(B)-\pi_{i}(A)-q(\pi_{i}(B)-\pi_{S})} \end{array}$$ (8)

Accordingly, we obtain that *p*_AC,P50_ > *p*_AC,P100_ for all *q* > 0 since *π*_*i*_(A) > *π*_*i*_(C) > *π*_S_ as shown in Eq (9) and, hence, that the *p*-range in which sending a payoff-equalizing message dominates is also wider in P50 than in P100.

$$\begin{array}{l} p_{AC,P50}=\frac{\pi_{C}-\pi_{\bar{A}}}{\pi_{\bar{C}}-\pi_{\bar{A}}+\pi_{i}(A)-\pi_{i}(C)+0.5q(\pi_{i}(C)-\pi_{S})}\\ >p_{AC,P100}=\frac{\pi_{\bar{C}}-\pi_{\bar{A}}}{\pi_{\bar{C}}-\pi_{\bar{A}}+\pi_{i}(A)-\pi_{i}(C)+q(\pi_{i}(C)-\pi_{S})} \end{array}$$ (9)

Regarding the effectiveness of punishment with different detection probabilities, we conclude that senders are expected to be honest for smaller *p*-ranges in P50 compared to P100 if *q* > 0, independently of the payoff scenario and the amount to which the sender's earnings are reduced by punishment, as long as *π*_S_ < *π*_i_(*z*). Since this effect is even more pronounced when comparing P100 to P25 and P10, respectively, we hypothesize that the rate of honest messages will decrease with the probability of detecting a lie.

***H2***: *Senders will be more likely to send honest messages the higher the probability of detecting a lie*.

With regards to the different payoff scenarios in each treatment, we will use scenario 1 as a basis for comparison. It is straightforward to show that the *p*-range in which truth-telling dominates is comparatively smaller in scenario 3 than in scenario 1 for the same value of *q* since senders can simply obtain a much higher payoff from deceiving his counterpart in scenario 3. In Table A in S1 Appendix, we present the respective ranges of *p* applied to the scenario-specific payoffs in the P-treatments. This continues to be true if we allow for the possibility of the sender assigning different *q*_*i*_ to different payoff scenarios. In general, the influence of the different payoff scenarios on the senders' expected *q* should be very limited since we do not provide receivers with information about the payoffs in the non-selected scenarios. Hence, we hypothesize that the higher the potential payoff from deception, keeping the receivers' relative payoffs constant, the less inclined senders will be to send honest messages when punishment is possible.

***H3***: *In the P-treatments, senders will be more likely to send honest messages in scenario 1 compared to scenario 3*.

Besides the different financial temptations among the scenarios, we are also interested in the sender's sensitivity to the harm he causes to his counterpart with successful deception under the punishment mechanism. We know from previous studies such as [17] that individuals take into account the negative consequences of deception for others, which can be represented by higher costs of lying *L*_i_ in scenario 2 than in scenario 1. Hence, when we give up our simplicity assumption of zero costs of lying, this would lead to a wider *p*-range in which honesty becomes a dominant strategy in scenario 2 compared to scenario 1. Furthermore, if a sender adjusts *q*_*i*_ among the scenarios, it is reasonable to assume that he assigns *q*_*i*_ a higher value in scenario 2 than in scenario 1, due to the receiver's higher relative loss in scenario 2. The adjustment of *q*_*i*_ would lead to an even wider *p*-range in which honesty dominates in scenario 2 compared to scenario 1. Therefore, we hypothesize that a sender is less inclined to lie when deception causes a greater harm to his counterpart in the presence of punishment possibilities.

***H4***: *In the P-treatments, senders will be less likely to send honest messages in scenario 1 compared to scenario 2*.

### Receivers

We turn now to the analysis of receiver behavior by asking whether these subjects trust their counterparts more often when they are able to punish them for dishonesty compared to a situation without the possibility of sanctioning. Receivers are blind regarding payoffs in our design and so we are not able to apply a similar subjective equilibrium analysis to this player type. The receiver's decision depends on her beliefs about the probability *r* ∊ [0,1] with which her counterpart has sent an honest message. If the receiver expects *r* to be greater than 0.5, she maximizes her expected payoffs by accepting this message and vice versa in T100. A receiver is indifferent between accepting and rejecting a message in case *r* = 0.5. We hypothesize that receivers will anticipate the deterrence effect of potential punishment predicted by our analysis, i.e., that receivers will show more trust in the senders’ message when they are able to punish dishonest senders than in the sanction-free baseline.

***H5***: *Receivers are more likely to accept a message in P100 than in T100*.

In line with our predictions of sender behavior, we further expect that receivers will anticipate a lower rate of honest messages when the probability of disclosing dishonesty decreases.

***H6***: *Receivers are less likely to accept a message with a lower probability of detecting a lie*.

## Results and discussion

### Sender behavior

This section reports sender behavior in terms of their message selection per treatment and payoff scenario. In our analysis we mainly focus on the rate of honest messages since deceptive and payoff-equalizing messages can both be characterized as dishonest and, when combined, simply represent the counterpart of honest sender behavior with respect to the full number of sender observations per treatment. Recall that receivers can reduce a dishonest sender’s final payoff to 2€ without bearing any costs when punishment is possible in the P-treatments.

**Fig 3:**
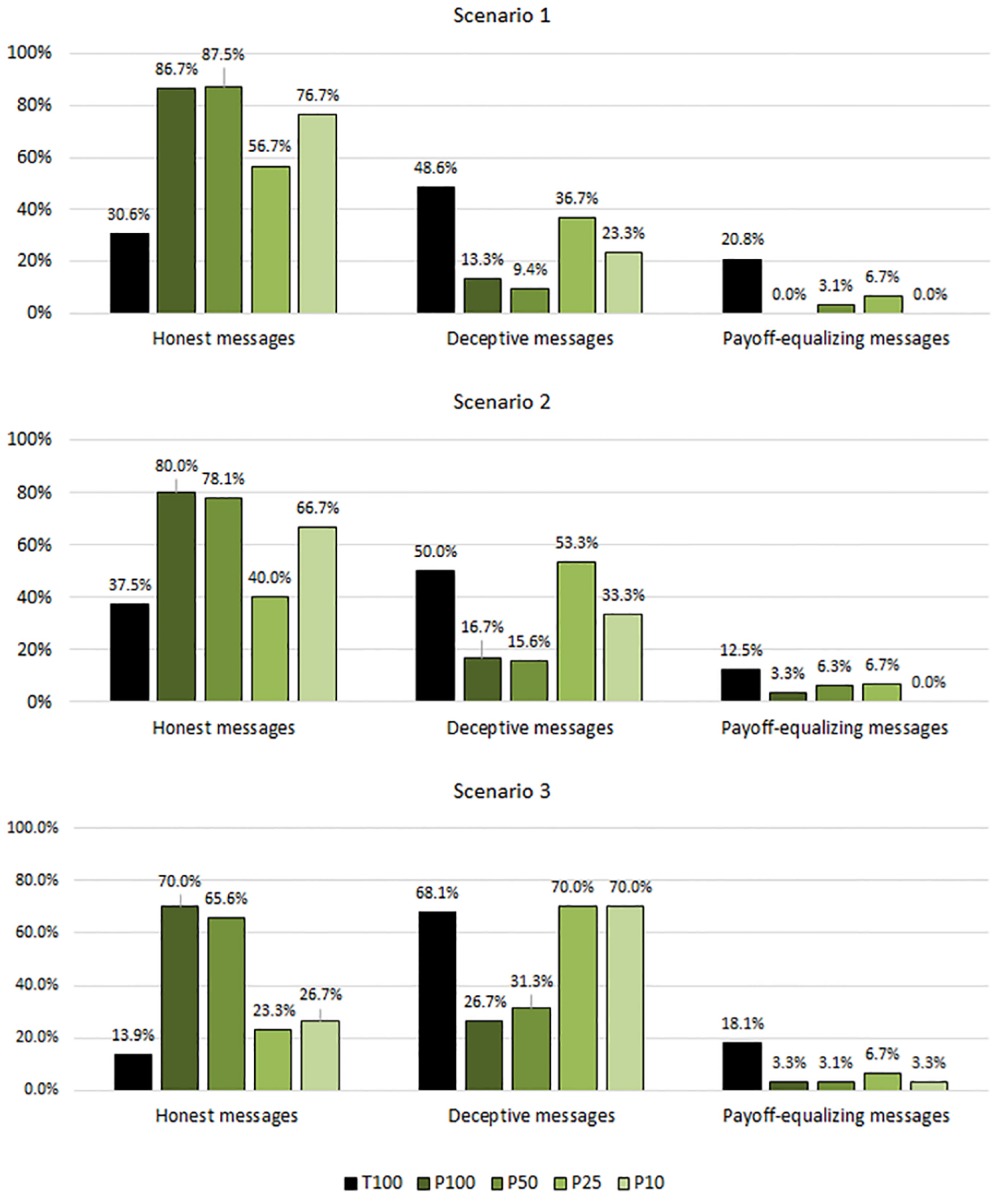
Fractions of messages chosen by senders across treatments and scenario.

Our main results are depicted in Fig 3. In contrast to many other punishment calibrations that were experimentally tested in the deception domain, we find that the presence of a severe and cost-free sanction system enhances pro-social behavior substantially. While, in scenario 1, 30.6% of the senders selected an honest message in T100, the rate of honest messages almost triples to 86.7% in P100. Similar differences are observable in scenario 2 (37.5% in T100 vs. 80.0% in P100) and scenario 3 (13.9% in T100 vs. 70.0% in P100). Chi^2^ proportion tests show that the rates of honest messages are significantly higher in P100 than in T100 (*p*<0.001 in all three scenarios). Hence, we find strong support for our hypothesis H1.

***Result 1***: *In the case of assured revelation of sender behavior, senders select honest messages significantly more often when dishonest behavior can be punished*.

We turn now to the question of how a variation in the probability of detecting a lie influences the deterrence effect of our punishment mechanism. Therefore, we compare the rates of honest messages among our four P-treatments. According to our expected utility analysis, punishment possibilities should lead to a lower rate of honest messages when the probability of revealing sender behavior is lowered.

By contrast, we do not observe a significant difference in the fractions of honest messages in any payoff scenario when the detection probability of P100 is halved in P50 (*p* = 0.922 in scenario 1, *p* = 0.856 in scenario 2, *p* = 0.713 in scenario 3). Strikingly, the same is true for scenario 1 (*p* = 0.317) and scenario 2 (*p* = 0.243) when we compare P100 with P10, in which the detection probability is substantially reduced by 90 percentage points. Only in scenario 3, where senders can gain the highest payoff from the game, we find a significantly lower rate of honest messages in P10 than in P100 (*p* = 0.001).

Our analysis of sender behavior indicates that, by using severe and cost-free punishment, monitoring can be considerably reduced while maintaining high levels of honesty. Therefore, we cannot confirm our hypothesis H2. However, the relationship between detection probabilities and honesty does not follow a linear trend since we observe that the rate of honest messages in P25 is significantly lower than in P100 in all payoff scenarios (*p* = 0.010 in scenario 1, *p* = 0.002 in scenario 2, *p*<0.001 in scenario 3).

***Result 2***: *With severe and cost-free punishment, monitoring can be considerably reduced while maintaining high levels of honesty. However, the relation between monitoring and honesty does not follow a linear trend and becomes sensitive to the senders’ potential stakes when the detection probability is at a low level*.

Our comparison of different payoff scenarios, with scenario 1 as the basis, shows that the rates of honest messages are lowest in scenario 3, in which the sender can gain a comparatively higher amount from deception. According to a McNemar test, which we use for this within-subject comparison, the differences between scenario 1 and scenario 3 are statistically significant in P50 (*p* = 0.035), P25 (*p* = 0.004) and P10 (*p* = 0.001). The difference in P100 is marginally significant (*p* = 0.096). These results confirm our H3 in the sense that our punishment mechanism is less effective in reducing deception when the stakes for senders are relatively high, in line with the scenario comparison in [17].

On the other hand, we do not find significant differences between scenario 1 and scenario 2, except for a marginal significance with *p* = 0.096 in P25 (*p* = 0.476 in P100, *p* = 0.257 in P50, *p* = 0.317 in P10). Therefore, we cannot confirm our H4. Like in scenario 1, senders can achieve only a small profit from deception in scenario 2 but at the expense of a much higher comparative loss for their counterpart. Therefore, deception can be characterized as “mean” in scenario 2. However, senders seem to expect a similar frequency of punishment in both scenarios, independently of the relatively larger harm to the receiver.

***Result 3***: *When punishment is possible, senders choose honest messages relatively less often when they can gain a comparatively higher amount from deception. However, their decisions are not significantly affected by a variation in the negative consequences of deception for their counterpart*.

We observe a broad range of deceptive messages between 9.4% and 70.0% depending on the treatment and payoff scenario. In the three treatments with a detection probability of less than 100% it is possible that risk-loving subjects take into account the possibility of not being revealed in the end of the game and therefore decide to deceive their counterpart. However, this strategy would not explain the positive rates of deceptive messages up to 26.7% in P100, where the revelation of deception is inevitable. We assume that these subjects do not expect to be punished either because of a general lack of interest in sanctioning by their counterpart or a possible discomfort the receiver may experience when reducing the sender's payoff to a very low level. Let us recall that sanctioning does not entail any financial cost for the receiver but also no financial gain. As we describe in section 5.3, a substantial fraction of receivers is indeed not punishing senders although being in the position to enforce a sanction.

With regard to payoff-equalizing messages, our subjective equilibrium analysis predicts that in treatments without punishment, sending this message type is a dominant strategy for risk-neutral senders who expect their counterparts to trust them with a subjective acceptance probability of *p* < 0.33. Accordingly, payoff-equalizing messages are frequently selected by senders in T100 with up to 20.81% maximum frequency. On the other hand, this message type nearly disappears in our treatments with punishment. We find the highest rate, 6.7%, in the three scenarios of P25. In scenario 1 of P100 as well as in scenarios 1 and 2 of P10 none of the senders selected payoff-equalizing messages.

### Sender beliefs

**Fig 4:**
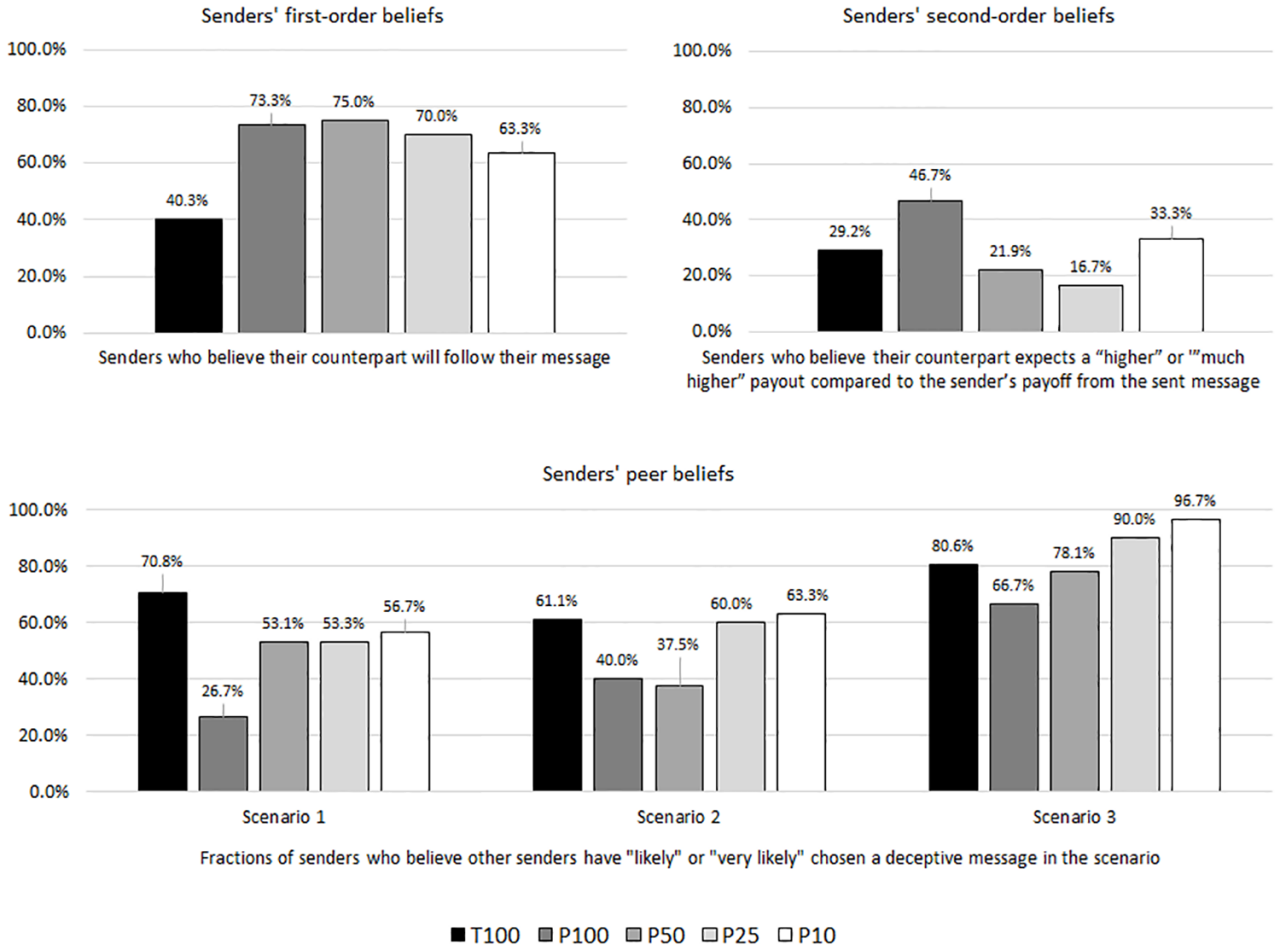
Senders’ beliefs across treatments.

In the following, we examine the role of senders’ beliefs (see section 3.3) as moderators of their behavior. The distributions across treatments are depicted in Fig 4. We begin with senders’ first-order beliefs about whether or not the receiver will implement the option mentioned in the sent message. While only 40.3% of the senders expect their counterpart to follow their message in the sanction-free baseline T100, this fraction increases substantially in all P-treatments, ranging from 63.3% to 75.0%. According to a chi^2^ test, the difference between T100 and P100 (40.3% vs. 73.7%) is significant with *p* = 0.002, indicating that relatively more senders expect their receiver to trust them when they have the possibility to punish deceptive behavior.

Moreover, the respective differences between P100 and the P-treatments with lower detection probabilities are not significant (*p* = 0.881 for P50, *p* = 0.774 for P25, *p* = 0.405 for P10). This is largely in line with the robust effect of our punishment mechanism on senders’ honesty that we reported above. It is also consistent with our finding that receivers actually show more trust independently of the detection probability when punishment is possible, as presented in section 5.3.

***Result 4***: *Senders anticipate an increased acceptance rate by receivers when punishment is possible, independently of the detection probability*.

Second-order beliefs vary moderately across treatments. We find the highest fraction of senders, who believe that their counterpart expects a “higher” or “much higher” payout compared to their own one, in P100 (46.7%). The difference to the respective fraction in T100 (29.2%) is marginally significant with *p* = 0.090 and in line with the fact that a higher fraction of senders expects their counterparts to trust them in the presence of the punishment mechanism. Significantly lower fractions of senders believe that their counterpart expects a relatively higher payoff in P50 than in P100 (21.9%; *p* = 0.039) and also in P25 (21.9%; *p* = 0.012). Only in P10 the respective fraction is not significantly different from P100 (33.3%; *p* = 0.439). This finding is in line with the fact that a 10% detection probability leads to a strong deterrence effect of our punishment mechanism.

With respect to peer beliefs, we find a significant difference comparing T100 with P100 in scenario 1 (70.8% vs. 26.7%; *p*<0.001) and a marginally significant one in scenario 2 (61.1% vs. 40.0%; *p* = 0.051). In these scenarios, a substantially lower fraction of senders expects other players in their role to deceive their counterparts when there is a possibility of being punished. The difference is not significant in scenario 3 (80.6% vs. 66.7%; *p* = 0.132).

Regarding the different detection probabilities, the fraction of senders expecting their peers to deceive is significantly different between P100 and P50 in scenario 1 (26.7% vs. 53.1%; *p* = 0.034) but not in the other two scenarios. Moreover, these fractions are significantly higher in most of the scenarios of P25 and P10 compared to P100, indicating that relatively more senders expect their counterparts to deceive when the detection probability is reduced (P25: *p* = 0.035 in scenario 1; *p* = 0.018 in scenario 2. P10: *p* = 0.018 in scenario 1; *p* = 0.071 in scenario 2; *p* = 0.003 in scenario 3.). This tendency is most accentuated in scenario 3 in the lower right part of Fig 4.

**Fig 5:**
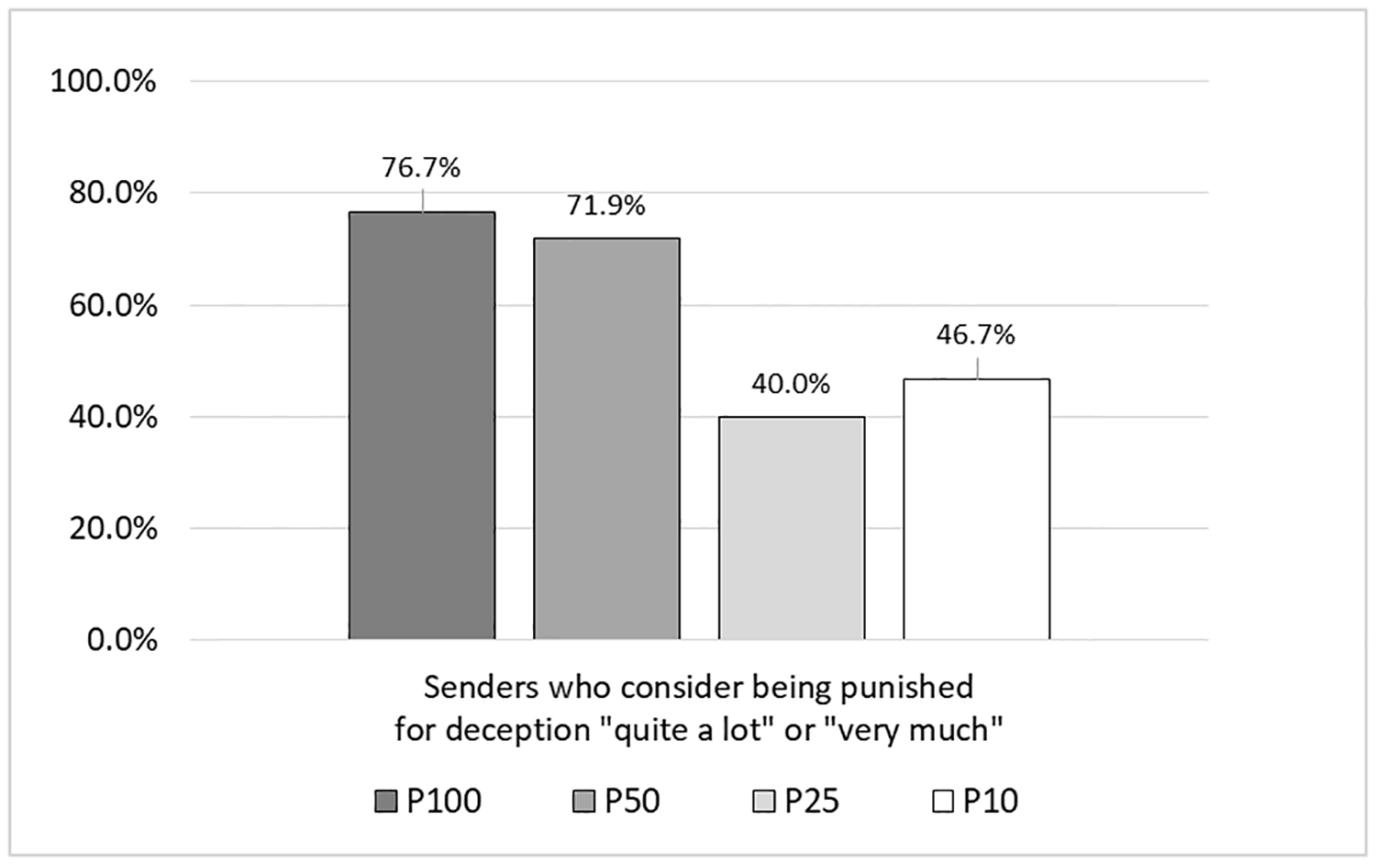
Senders’ punishment considerations across treatments.

Finally, we analyze senders’ punishment considerations. We find that the fraction of senders, who considered being punished by their counterpart in case they send a dishonest message “quite a lot” or “very much” in their decision, is slightly lower in P50 (71.9%) compared to P100 (76.7%). However, this difference is not significant (*p* = 0.667). When we reduce the detection probability further, the fractions decrease substantially to 46.7% in P25 and to 40.0% in P10. Compared to assured disclosure in P100, the differences are significant with *p* = 0.004 for P25 and *p* = 0.017 for P10. This tendency is intuitive as senders consider being punished less when the detection probability is reduced. The rates are depicted in Fig 5.

In order to investigate to which extent senders’ beliefs and punishment considerations affect individual behavior in our P-treatments, we use probit regressions for each payoff scenario with a dependent variable that takes the value “1” in case the sender chose to send an honest message, “0” otherwise. The sample comprises data from our four treatments with punishment mechanism and consists of 122 observations in each model (32 senders in P50 and 30 senders in the remaining P-treatments). The regression results are presented in Table 4. In models I-III, we include dummy variables for each P-treatment except for P100, which serves as our baseline in all models. In models IV-VI we add the senders’ first-order, second-order and peer beliefs as well as their punishment considerations. In models VII-IX we add socio-demographic factors as controls for gender, age, income, major, and siblings.

**Table 4:** Determinants of senders’ selection of honest messages.

	I	II	III	IV	V	VI	VII	VIII	IX
Scenario	1	2	3	1	2	3	1	2	3
P50	0.040	-0.065	-0.122	0.427	-0.073	0.042	0.577	-0.032	-0.065
	(0.405)	(0.360)	(0.332)	(0.458)	(0.378)	(0.347)	(0.502)	(0.401)	(0.378)
P25	-0.943**	-1.095***	-1.252***	-0.463	-0.988***	-1.030***	-0.454	-1.071***	-1.071***
	(0.369)	(0.349)	(0.349)	(0.433)	(0.378)	(0.370)	(0.455)	(0.387)	(0.379)
P10	-0.383	-0.411	-1.147***	0.029	-0.245	-0.984***	0.174	-0.273	-0.891**
	(0.383)	(0.352)	(0.344)	(0.430)	(0.372)	(0.373)	(0.461)	(0.383)	(0.380)
First-order beliefs				0.227	0.210	-0.435	0.188	0.161	-0.369
				(0.317)	(0.295)	(0.285)	(0.341)	(0.305)	(0.297)
Second-order beliefs				0.648*	0.209	0.332	0.734*	0.341	0.293
				(0.375)	(0.296)	(0.284)	(0.414)	(0.311)	(0.300)
Peer group deception				-1.037***	-0.750***	-0.678*	-1.135***	-0.724***	-0.771**
				(0.311)	(0.269)	(0.355)	(0.346)	(0.274)	(0.373)
Punishment considerations				0.574*	-0.037	0.123	0.757**	-0.107	0.159
				(0.303)	(0.271)	(0.269)	(0.338)	(0.279)	(0.278)
Constant	1.111***	0.842***	0.524**	0.761*	0.996**	1.089**	4.331***	2.098*	1.919*
	(0.288)	(0.261)	(0.241)	-0.423)	(0.396)	(0.438)	(1.414)	(1.204)	(1.124)
Further controls	No	No	No	No	No	No	Yes	Yes	Yes
Chi^2^	10.11	13.55	23.38	29.09	23.07	30.36	37.38	26.53	35.08
Pseudo R^2^	0.08	0.09	0.14	0.22	0.15	0.18	0.28	0.17	0.21

*** *p*-value < 0.01.

** *p*-value < 0.05.

* *p*-value < 0.1.

Standard errors in parentheses. N = 122.

We included the dummies P50, P25 and P10 that indicate whether the sender participated in one of the three treatments with reduced detection probabilities. The dummy “First-order beliefs” takes the value 1 when a sender expects his counterpart to follow the sent message. “Second-order beliefs” takes the value 1 when a sender believes his counterpart to expect a relatively higher payoff from the sent message. “Peer group deception” takes the value 1 when a sender believes that it is likely or very likely that other subjects in their role select deceptive messages in the payoff scenario. “Punishment considerations” takes the value 1 when a sender considers in his decision being punished by the receiver “quite a lot” or “very much”. We added further controls that include the senders’”Age” as well as the dummies “Female”, “Econ_bus” indicating whether the sender is studying economics, management or finance, “Funding” indicating whether the sender receives monthly earnings from a job or a university fellowship, and “Siblings” indicating whether the sender has one or more siblings. The detailed results of these further control variables are presented in Table B in S1 Appendix.

The coefficients of the treatment dummies confirm that the stable deterrence effect of punishment in our setting is robust when we control for individual beliefs and socio-demographic factors. There is no significant effect on selecting honest messages in any payoff scenario when the detection probability is comparatively reduced in P50. The same is true for senders participating in P10, except for the high stake scenario 3, in which selecting honest messages is significantly less probable compared to P100. As expected, the coefficients for P25 show that reducing the detection probability to 25% leads to significantly less honest messages selected by senders, confirming the non-linear relationship that we reported above. However, when controlling for senders’ beliefs and further factors, none of the reductions in detection probabilities has a significant negative effect on selecting honest messages in scenario 1 (see model VII).

In contrast to their important role for sender behavior in similar settings without punishment [18], senders’ first- and second-order beliefs do not show any effect at conventional levels in our P-treatments. On the other hand, peer beliefs have a large effect in terms of coefficients on senders’ behavior under the punishment mechanism. The relative probability of selecting an honest message is significantly lower when a sender expects other players in their role to deceive their counterpart in the respective scenario. We also do not find any significant interaction between peer beliefs and the different levels of detection probabilities (see Table B in S1 Appendix). Punishment considerations have a significant effect on senders’ decision only in scenario 1 when controlling for further factors, in the sense that a sender is more inclined to send an honest message the more he considers to be punished for being dishonest. Finally, in contrast to other findings in sender-receiver games, as in [64,65], we find no significant gender effects (see Table B in S1 Appendix). Moreover, we re-ran the regressions in specifications VII to IX of Table 4 including only one of the belief types at a time and obtained results that are similar to the ones reported above (see Table C and Table D in S1 Appendix).

***Result 5***: *The threat of a potential punishment and norm-related peer beliefs are the moderating factors for decisions between honest and dishonest messages on an individual level and this effect is largely independent of the detection probability*.

We turn now to an assessment of potential reasons for the stable deterrence effect of punishment that we observe in the P-treatments. According to our theoretical predictions, payoff-maximizing senders should exhibit a higher willingness to deceive with a reduced detection probability as their expected utility from sending deceptive messages increases in that case. Since we do not observe such higher willingness, other factors must exist that counterbalance the increase in expected utility from deceptive messages with reduced detection probabilities and, thus, keep senders from excessively selecting this type of messages.

Within our theoretical framework, there are two potential factors, related to strategic considerations, which could act as such a counterbalance: a decrease in the senders’ subjective probabilities of message acceptance *p* and an increase in their subjective probabilities of being punished for dishonesty *q*. A decreasing probability of message acceptance *p* seems plausible as receivers could potentially expect a lower rate of honest messages when they are less able to detect and punish dishonest senders. However, recall that we do not find a significant difference in the fractions of senders, who expect their counterpart to follow their message, among the four P-treatments, expressed by their first-order beliefs. This proves that there is no change in the average *p*, no matter to what extent we reduced the detection probability. Recall further that we observe substantially lower fractions of senders who consider the possibility of being punished in their decision with reduced detection probability. This finding indicates that one cannot assume an increase in the senders’ subjective probability of being punished *(q)* with reduced detection probabilities.

Alternatively, one could assume that in the P-treatments only those senders who do not expect to be punished by their counterparts select deceptive messages, and would therefore not be affected by a change in detection probabilities. But we find that a substantial fraction of senders in our experiment who deceived their counterpart in P100 and P50 and actually considered in their decision the possibility of being punished. This fraction varies between 13.0% (scenario 1 in P100) and 87.0% (scenario 1 in P50).

Another possibility would be that only those subjects who consider the possibility of being punished by their counterparts are honest in the P-treatments, and thus abstain from deception independently of the change in detection probabilities. In our setting, we also find heterogeneity among the honest senders with regards to punishment considerations as between 14.3% (scenario 3 in P10) and 91.7% (scenario 1 in P25) of the senders were honest in the P-treatments although they did not take into consideration the possibility of being punished.

Since no strategic components change among the P-treatments in a way that could explain the stable deterrence effect with lowered detection probability, we argue that the main driving force behind it can only be attributed to anticipated psychological costs of being punished for deceiving others. This explanation is in line with [2] as well as [10] who find that punished subjects indeed suffer from negative emotions related to the experienced sanction. Furthermore, it seems as if such psychological costs are not related to guilt-aversion. Second-order beliefs vary significantly between the P-treatments, indicating that, when the detection probability decreases, comparatively fewer senders believe that their counterparts expect relatively high payoffs from the message. Also, when comparing the social norm-related peer beliefs within each treatment family, we only find a significant difference between the punishment treatments in scenario 1 (*p* = 0.034), implying that other senders are more often expected to deceive when the probability of detection is only 50%. Beliefs about perceived social norms among peers are rather related to emotional motivations and have only limited relevance for strategic considerations resulting in scenario-specific effects.

***Result 6***: *The overall high levels of honesty in the P-treatments cannot be attributed to a change in strategic components such as the senders’ first-order beliefs or punishment considerations, leaving anticipated psychological costs of being punished for deception as a potential explanation*.

### Receiver behavior

**Fig 6:**
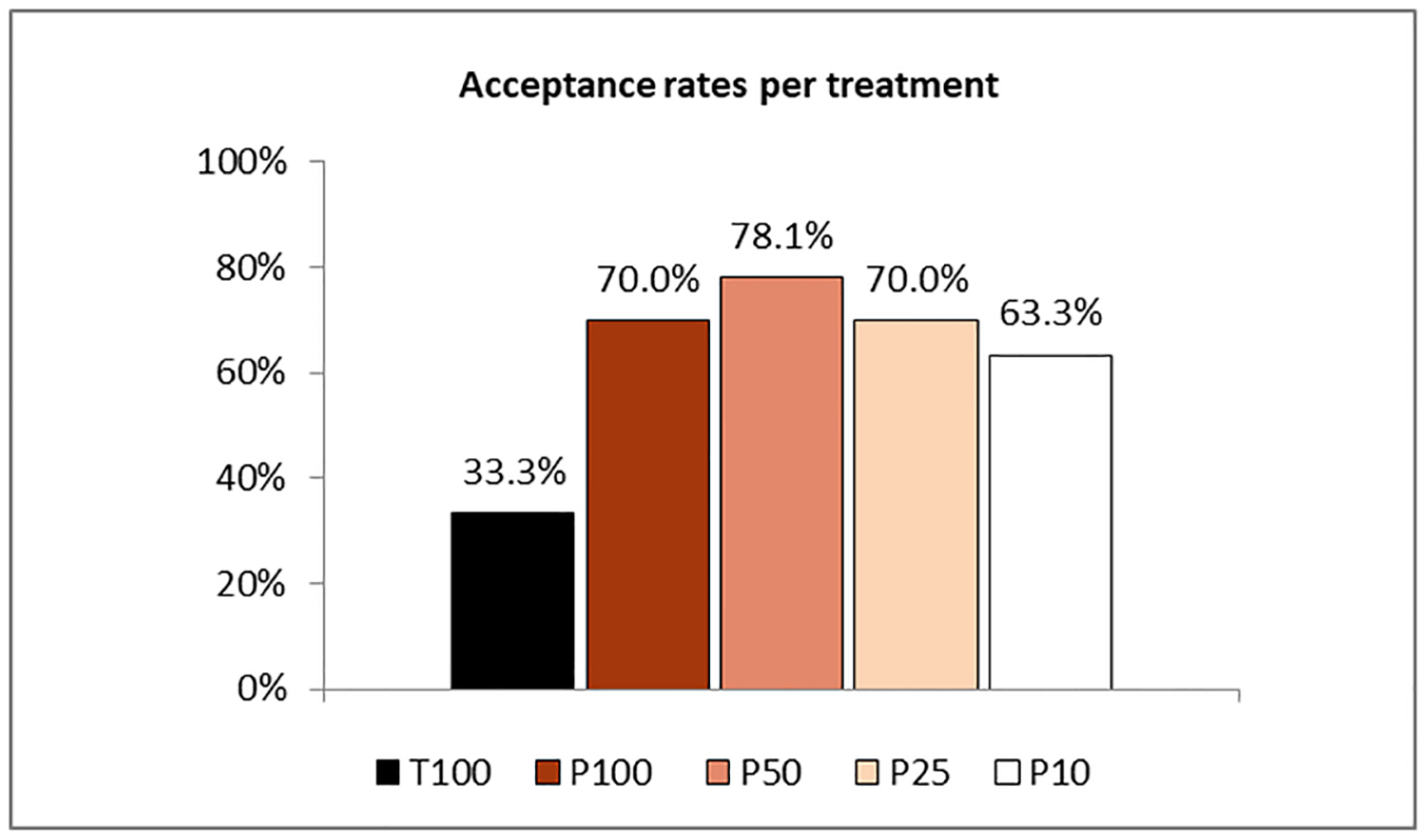
Receivers’ acceptance rates across treatments.

Receivers take on an active role in our experiment by deciding whether the option mentioned in the senders’ message is implemented or not. Since they do not learn the payoffs from each option in the beginning, accepting a message can be considered as an act of trust and rejection as an act of distrust towards the sender. Therefore, a comparison of the message acceptance rates across treatments, as presented in Fig 6, allows us to make a statement about how our punishment mechanism and the variation in the detection probability affects trust in our setting.

As expected, we find that the possibility to punish dishonest senders increases receivers’ trust substantially. While only 33.3% of the receivers accept the senders’ messages in our baseline T100, this fraction increases significantly to 70.0% in P100 according to a chi^2^ test (*p* = 0.001). Many receivers seem to correctly anticipate the strong effect that the threat of efficient punishment has on sender behavior. Hence, we can confirm H5.

Strikingly, and in line with the stable deterrence effect of our punishment mechanism across most treatments and payoff scenarios, we observe similarly high trust levels among receivers when the detection probability is reduced. The differences between P100 and the remaining P-treatments are not significant (*p* = 0.465 for P50; *p* = 1.000. for P25; *p* = 0.584 for P10). Thus, we cannot confirm our H6. We conclude that receivers seem to anticipate that punishment provokes a substantial deterrence effect independently of the detection probability, supporting our notion that monitoring effort could be reduced in our punishment setting while maintaining the levels of honesty and trust.

***Result 7***: *Receivers trust their counterparts significantly more often when they are able to punish dishonest senders and show similarly high trust levels independently of the detection probability*.

Due to the fact that the vast majority of senders selected honest messages in the P-treatments, only few receivers had the possibility to sanction their counterpart for a dishonest message they accepted. The respective rates of possible punishments vary from 6.7% in P10 to a maximum of 18.8% in P50, as shown in Table 5. Interestingly, we find that only two out of four possible punishments in P100 are enforced. The enforcement rates are even lower in P25 (33.3%) and P50 (16.7%), a fact that does not seem to be anticipated by senders. Out of the seven times when punishment was enforced, one time this happened in scenario 1, two times in scenario 2 and four times in scenario 3. While these findings have to be interpreted with caution given the overall low number of punishments, it is surprising that so few receivers actually sanctioned their counterparts given that they did not have to bear any monetary costs in this anonymous setting. We assume that some receivers may rather face psychological costs of severely punishing their dishonest counterparts and, hence, are reluctant to actually enforce the sanction.

**Table 5:** Punishment possibilities and enforcement rates.

Treatment	Possibilities to punish	Enforced punishments
P100	4 (13.3%)	2 (50.0%)
Scenario 1	0	0
Scenario 2	3	1
Scenario 3	1	1
P50	6 (18.8%)	1 (16.7%)
Scenario 1	1	0
Scenario 2	1	0
Scenario 3	4	1
P25	3 (10.0%)	2 (66.7%)
Scenario 1	0	0
Scenario 2	1	0
Scenario 3	2	2
P10	2 (6.7%)	2 (100.0%)
Scenario 1	1	1
Scenario 2	1	1
Scenario 3	0	0

### Receiver beliefs

**Fig 7:**
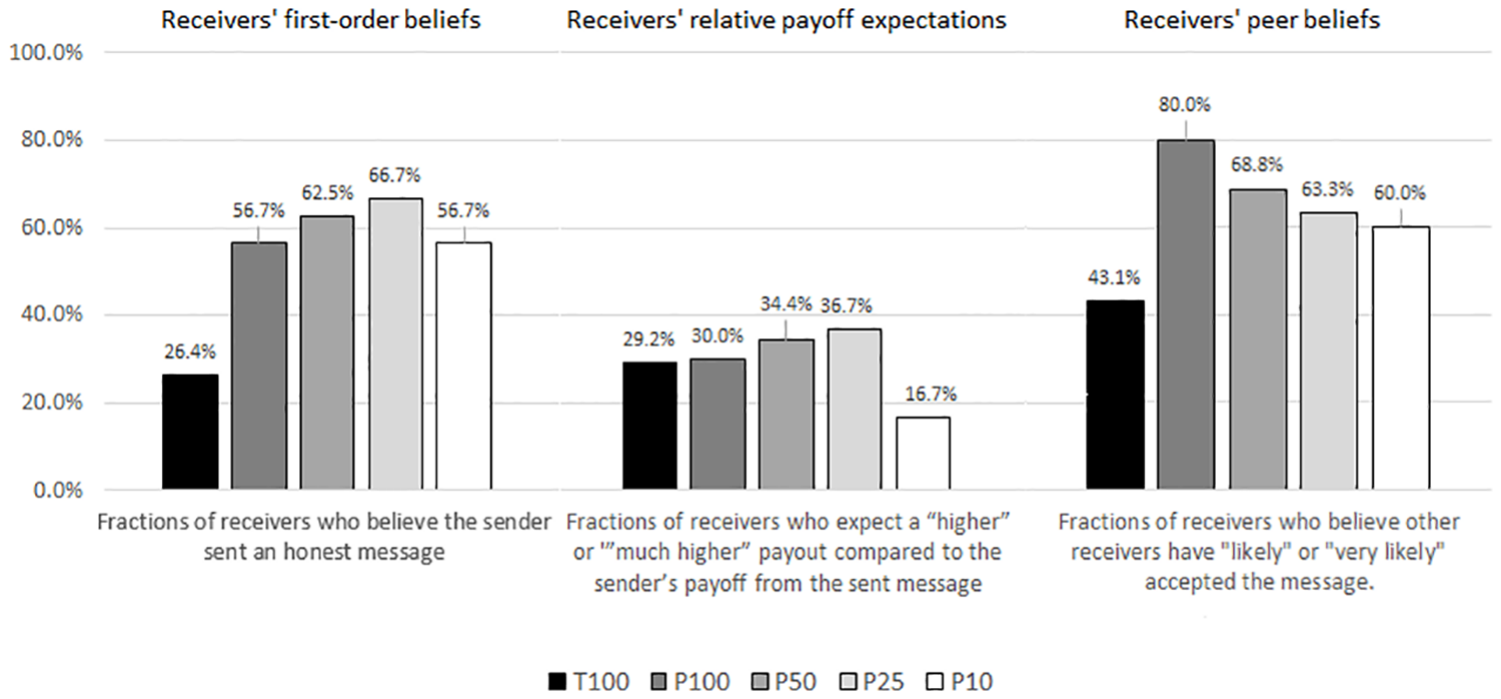
Receivers’ beliefs across treatments.

Fig 7 depicts the belief-related fractions of receivers. According to a chi^2^ test, first-order beliefs are significantly different between T100 and P100 (*p* = 0.004), in the sense that more receivers expect senders to send an honest message when punishment is possible. In line with the stable trust across our P-treatments, this fraction does not change significantly when the detection probability is lower than in P100 (*p* = 0.640 for P50; *p* = 0.426 for P25; *p* = 1.000 for P10).

Moreover, the fraction of receivers who expect to gain more than the sender from the option mentioned in the message does not change significantly from T100 to P100 (*p* = 0.933). Although this fraction is noticeably lower than in P100 when the detection probability is at its lowest level in P10, the differences among P100 and the other P-treatments are not significant (*p* = 0.713 for P50; *p* = 0.584 for P25; *p* = 0.222 for P10). In line with our previous findings, comparatively more receivers believe that the other players in their role will accept the message in P100 compared to the sanction-free T100 (*p* = 0.001). Again in line with the stable trust across the P-treatments, this fraction of receivers does not exhibit a significant change at conventional levels when the detection probability is reduced, independently of the detection probability (*p* = 0.312 for P50; *p* = 0.152 for P25; *p* = 0.091 for P10).

We use probit regressions to assess the determinants of receiver behavior in our P-treatments. As in the case of sender beliefs (Table 4), the sample comprises data from our four treatments with punishment mechanism, consisting of 122 observations in each model. The dependent variable takes the value 1 when a receiver accepts the sender's message, that is, the option mentioned in the message determines the payoff of both players. We present the respective coefficients in Table 6. In model I we include dummy variables for each P-treatment except for P100, which serves as our baseline, model II adds receivers’ individual beliefs and model III includes further controls (gender, age, major, income, and siblings). In line with the stable acceptance rates across the P-treatments, we do not find a significant effect on trust when being in a treatment with a lower detection probability than in P100, controlling for beliefs and further factors.

**Table 6:** Determinants of receivers’ acceptance of messages.

	I	II	III
P50	0.252	0.544	0.299
	(0.345)	(0.538)	(0.616)
P25	0.000	-0.122	-0.544
	(0.340)	(0.603)	(0.762)
P10	-0.184	-0.443	-1.072
	(0.335)	(0.557)	(0.668)
First-order beliefs		2.983***	4.113***
		(0.583)	(0.999)
Relative payoff expectations		-1.134**	-1.779**
		(0.509)	(0.744)
Peer group trust		0.945**	0.871*
		(0.419)	(0.470)
Constant	0.524**	- 0.796*	2.398
	(0.241)	(0.463)	(1.962)
Further controls	No	No	Yes
Chi^2^	1.67	95.19	102.27
Pseudo R^2^	0.01	0.64	0.69

*** *p*-value < 0.01.

** *p*-value < 0.05.

* *p*-value < 0.1.

Standard errors in parentheses. N = 122.

In addition to the treatment dummies P50, P25 and P10, we added the dummy “First-order beliefs” that takes the value 1 when a receiver expects her counterpart to have sent an honest message. The dummy “Relative payoff expectation” takes the value 1 when a re**ce**iver believes her counterpart to expect a relatively higher payoff from the sent message. “Peer group trust” takes the value 1 when a receiver believes that it is likely or very likely that other subjects in their role accept the received message. Further controls include the receivers’”Age” as well as the dummies “Female”, “Econ_bus” indicating whether the receiver is studying economics, management or finance, “Funding” indicating whether the receiver receives monthly earnings from a job or a university fellowship, and “Siblings” indicating whether the receiver has one or more siblings.

Interestingly, and in contrast to senders, individual beliefs are the main determinants of receiver behavior in our study. In terms of coefficients, the first-order beliefs show the largest effect size. It seems intuitive that receivers are more likely to accept a message when they expect senders to be honest. The negative coefficients of relative payoff expectations can be attributed to the tendency of selfish receivers to distrust their counterparts, as mentioned in [18]. Furthermore, receivers are more likely to trust a sender when they expect their peers to accept the messages they received. Interestingly, we find that female receivers show significantly less trust in the senders’ message than male receivers (see Table E in S1 Appendix).

***Result 8***: *Overall, the punishment effect on trust is mediated by receivers’ individual beliefs. Furthermore, female receivers are less prone to trusting the messages*.

## Conclusion

In this study, we established a well-functioning punishment system for deterring deception in principal-agent relationships. Altogether, our findings indicate that punishment works in a particular way in the deception context, which should be taken into account in the development of efficient measures against economic fraud: In contrast to many other punishment calibrations tested in the literature, a highly efficient punishment mechanism proves to be a credible threat for potential deceivers.

Importantly, the punishment mechanism we apply does not require complete monitoring to be effective. While a subjective equilibrium analysis predicts that our mechanism should lead to a lower rate of honest messages when the probability of detecting a lie is significantly reduced, we do not observe such differences. By analyzing subjects' beliefs, we can rule out a change in strategic considerations of senders and suggest that the stable deterrence is observed due to anticipated psychological costs of being punished for deception.

An exception is one treatment with a moderate detection probability, where the rates of honest messages are significantly lower than in the case of assured revelation. We conclude that the relationship between monitoring and honesty does not follow a linear trend as moderate monitoring proves to be less effective in enhancing honesty than high or low monitoring levels. Although it is necessary to account for this non-linearity, our results suggest that monitoring costs in principal-agent relationships could be considerably reduced without risking a major decline in honesty. In addition, this implication is particularly important regarding the existence of thresholds observed in the literature (for instance, [24]) above which monitoring is able to crowd-out norm obedience.

By using a within-subject comparison of different payoff scenarios, we confirm that the deterrence effect of punishment is less pronounced when senders face the temptation of gaining a comparatively high amount from deception. However, senders seem to be unaffected by the financial consequences of deception in terms of the receiver's relative loss when facing the possibility of being sanctioned.

Highly efficient punishment does not only affect senders’ honesty in a positive way but also leads to substantially higher trust levels among receivers. This is particularly striking, since we observe this behavior in a setting without repeated interactions and, hence, without the possibility of reputation building. Moreover, the finding is an important insight since many economic relationships are unique and an intact economic relationship can only be established when all involved parties actually agree to interact with each other. Even a perfectly working deterrence mechanism is not an optimal tool if it does not convince individuals to establish economic relationships in the first place. Furthermore, there are no significant differences between the message acceptance rates in the punishment treatments, implying that receivers correctly anticipate the robust deterrence effect when detection probabilities are reduced to a minimum level. However, receivers are excessively optimistic in the sense that they do not anticipate the relation between honesty and detection probabilities to be non-linear.

Overall, we find surprisingly low enforcement rates. Since sanctioning was cost-free and not-profitable to the enforcer in our design, we assume that the majority of receivers were reluctant to punish because of an emotional discomfort resulting from the severity of the sanction. Our results might be an indication for the act of punishing being not only linked to emotions like anger about the unethical act or guilt for otherwise forgone opportunities to punish (see [66]), but also to pity in terms of an anticipation of anger and guilt that the punished person would experience [10]. This might result in opposing motivations of the involved party with respect to the enforcement of punishment as in [67], based on the severity of the sanction in relation to the size of the norm defection.

Given that we fixed the detection probabilities and the punishment level exogenously, an additional potential for further investigation would be to allow for an endogenous monitoring level or an endogenous punishment level. As shown in the model of [68], the deterrence effect of punishment can even decrease when a central authority delegates the monitoring task to an autonomous agent with own interests. Furthermore, the introduction of repeated interactions with sanctioning possibilities, as in [35], could cast some light on the stability of the deterrence effect of cost-free punishment and on the sanction enforcement rates under different detection probabilities in the long run.

## Supporting information

S1 Appendix Experiment instructions, additional calculations and additional regressions. Table A. Value ranges of p in which honesty is the dominant strategy in the P-treatments. Table B. Determinants of senders’ selection of honest messages including further controls (models I-III) and interactions of treatments with peer beliefs (models IV-VI). Table C. Determinants of senders’ selection of honest messages including first-order beliefs (models I-III) and second-order beliefs (models IV-VI). Table D. Determinants of senders’ selection of honest messages including peer beliefs (models I-III) and punishment considerations (models IV-VI). Table E. Determinants of receivers’ acceptance of messages including further controls.
(DOCX)
