# Supplementary material for: Punishing liars—How monitoring affects honesty and trust
Source: PLoS One. 2018 Oct 10;13(10):e0205420. doi: 10.1371/journal.pone.0205420 (PMC6179269; doi:10.1371/journal.pone.0205420)
Supplement: S1 Appendix — Table A. Value ranges of p in which honesty is the dominant strategy in the P-treatments. Table B. Determinants of senders’ selection of honest messages including further controls (models I-III) and interactions of treatments with peer beliefs (models IV-VI). Table C. Determinants of senders’ selection of honest messages including first-order beliefs (models I-III) and second-order beliefs (models IV-VI). Table D. Determinants of senders’ selection of honest messages including peer beliefs (models I-III) and punishment considerations (models IV-VI). Table E. Determinants of receivers’ acceptance of messages including further controls. (DOCX) [file pone.0205420.s001.docx]

**Appendix for “Punishing liars – how monitoring affects honesty and trust”**

**Sascha Behnk, Iván Barreda-Tarrazona, Aurora García-Gallego**

**A1. Experiment instructions**

(translated from Spanish)

Welcome to this experiment, we greatly appreciate your participation. From this moment on, please switch off your cell phone and do not talk or communicate in any way with the other participants. Read these instructions carefully and raise your hand if you have any questions during the session. One of the officials of the experiment will answer your questions individually.

Your decisions in this experiment will allow you to earn a certain amount of money that we will pay you in cash at the end of the session.

You will be a player in a two-player game. Your partner will be one of the participants in this session, randomly assigned by the computer. None of you will know the identity of the partner at any time. One of you will be assigned the role of "Player 1" and the other the role of "Player 2".

You will interact with your partner only once and this will take place through the computers. After this interaction, the experiment will end and you will be asked to fill in a short questionnaire.

**Decision Making Player 1**

During the experiment we will present three scenarios to Player 1, each one them contains three options. Each option consists of a payoff for Player 1 and a payoff for Player 2. This is the general structure of the options in each scenario that will be presented to Player 1:

Option A: Player 1 receives ... euros and Player 2 receives ... euros.

Option B: Player 1 receives ... euros and Player 2 receives ... euros.

Option C: Player 1 receives ... euros and Player 2 receives ... euros.

We will present to Player 1 the payoffs for both players of each option and in each scenario (the order of the options is at random). By contrast, Player 2 will not get this information. Player 1's task is to choose one of the following three messages that will be sent to Player 2 afterwards:

Message 1: Option A will earn you more money than the other two options.

Message 2: Option B will earn you more money than the other two options.

Message 3: Option C will earn you more money than the other two options.

Remember that there are three scenarios. That means, Player 1 has to decide, in each scenario, which message she wants to be sent to Player 2.

After Player 1 has chosen a message for each scenario, the computer will randomly select one of the scenarios. This scenario will then be implemented and the specific message that Player 1 chose for this scenario will be sent to Player 2. From this moment on, it depends on the decision of Player 2 which of the three corresponding options will be implemented and, according to this, which amount of money both players will earn.

**Decision Making Player 2**

Player 2 knows about the three options in the selected scenario but she knows nothing about the earnings associated with each option. The only information that Player 2 receives is the message that Player 1 chose for the implemented scenario.

After receiving player 1’s message, Player 2 takes her decision, which is either to "accept" or "reject" the message. To "accept" the message means that Player 2 accepts the information of the message and that the option mentioned in the message determines the earnings of the two players. On the contrary, to "reject" the message means that Player 2 does not want the option mentioned in the message but another option to determine the earnings of both players. Therefore, if Player 2 accepts the message, the option in the message will be implemented and determines the payoffs of the players. In the case that Player 2 rejects the message, one of the remaining options of the selected scenario will be randomly implemented by the computer in order to determine the earnings of both players.

**Earnings**

Before your earnings are shown on the screen, you will answer some short questions. After that, Player 1 will receive information about the acceptance or rejection of her message, the implemented option corresponding to the scenario that was selected by the computer and the earnings of both players.

[***Treatment T100****:*

*Player 2 will receive information about her own earnings corresponding to the implemented option. Furthermore, Player 2 will receive information about all potential payoffs for both players of each options in the scenario that has been implemented.*]

[***Treatment P100****:*

*Player 2 will receive information about her own earnings corresponding to the implemented option. Furthermore, Player 2 will receive information about all potential payoffs for both players of each options in the scenario that has been implemented.*

*If player 1 sent a false message that was accepted, Player 2 will then have the possibility to punish Player 1 by reducing his profits to 2 euros.*]

[***Treatment P50****:*

*In principle, Player 2 will only receive information about her own payoff corresponding to the implemented option. Furthermore, a possibility exists that the computer decides to provide additional information to player 2 about all potential payoffs for both players of each option in the implemented scenario. The probability of this happening is 50%.*

*If player 1 sent a false message that was accepted, Player 2 will then have the possibility to punish Player 1 by reducing his profits to 2 euros.*]

[***Treatment P25****:*

*In principle, Player 2 will only receive information about her own payoff corresponding to the implemented option. Furthermore, a possibility exists that the computer decides to provide additional information to player 2 about all potential payoffs for both players of each option in the implemented scenario. The probability of this happening is 25%.*

*If player 1 sent a false message that was accepted, Player 2 will then have the possibility to punish Player 1 by reducing his profits to 2 euros.*]

[***Treatment P10****:*

*In principle, Player 2 will only receive information about her own payoff corresponding to the implemented option. Furthermore, a possibility exists that the computer decides to provide additional information to player 2 about all potential payoffs for both players of each option in the implemented scenario. The probability of this happening is 10%.*

*If player 1 sent a false message that was accepted, Player 2 will then have the possibility to punish Player 1 by reducing his profits to 2 euros.*]

The final earnings will be presented on the last screen.

After that we will pay you anonymously and in cash the amount that corresponds to your final earnings in the game.

Do you have any questions about these instructions? If so, please raise your hand. If you do not have any questions, remain silent until you get instructions from the experimenter.

**A2. Additional calculations**

Under standard economic theory, a sender would never send honest messages in the T-treatments since the expected utility of sending honest messages is lower than the one from sending deceptive messages for all *p* > 0.33:

$$EU_{T}(A)<EU_{T}(B)$$

$$\Leftrightarrow p\pi_{i}(A)+(1-p)\frac{\pi_{i}(B)+\pi_{i}(C)}{2}<p\pi_{i}(B)+(1-p)\frac{\pi_{i}(A)+\pi_{i}(C)}{2}$$

$$\Leftrightarrow p>\frac{\frac{\pi_{i}(B)+\pi_{i}(C)}{2}-\frac{\pi_{i}(A)+\pi_{i}(C)}{2}}{\frac{\pi_{i}(B)+\pi_{i}(C)}{2}-\frac{\pi_{i}(A)+\pi_{i}(C)}{2}+\pi_{i}(B)-\pi_{i}(A)}\Leftrightarrow p>\frac{1}{3}$$

The expected utility of sending honest messages is lower than the one from sending payoff-equalizing messages for all *p* < 0.33:

$$EU_{T}(A)<EU_{T}(C)$$

$$\Leftrightarrow p\pi_{i}(A)+(1-p)\frac{\pi_{i}(B)+\pi_{i}(C)}{2}<p\pi_{i}(C)+(1-p)\frac{\pi_{i}(A)+\pi_{i}(B)}{2}$$

$$\Leftrightarrow p<\frac{\frac{\pi_{i}(A)+\pi_{i}(B)}{2}-\frac{\pi_{i}(B)+\pi_{i}(C)}{2}}{\frac{\pi_{i}(A)+\pi_{i}(B)}{2}-\frac{\pi_{i}(B)+\pi_{i}(C)}{2}+\pi_{i}(A)-\pi_{i}(C)}\Leftrightarrow p<\frac{1}{3}$$

Accordingly, *EU_T_(A)* = *EU_T_(B)* = *EU_T_(C)* in case *p* =$\frac{1}{3}$.

Value ranges of *p* in which honesty is the dominant strategy exist in the P-treatments with *π_i_(A)* = 5 euros, *π_1,2_(B)* = 6 euros, *π_3_(B)* = 15 euros, *π_i_(C)* = 3 euros and a reduction to *π_S_* = 2 euros in case of a sanction:

Table A. Value ranges of *p* in which honesty is the dominant strategy in the P-treatments.

| *p*-ranges | P100 | P50 |
| --- | --- | --- |
| Scenarios  1 & 2 | $\frac{1}{3+q}<p<\frac{1}{3-8q}$  in case $q<0.375$ | $\frac{1}{3+0.5q}<p<\frac{1}{3-4q}$  in case $q<0.75$ |
|  | $\frac{1}{3+q}<p$  in case $q>0.375$ | $\frac{1}{3+0.5q}<p$  in case $q>0.75$ |
| Scenario 3 | $\frac{1}{3+q}<p<\frac{1}{3-2.6q}$ | $\frac{1}{3+0.5q}<p<\frac{1}{3-1.3q}$ |

**A3. Additional regressions**

Table B. Determinants of senders’ selection of honest messages including further controls

(models I-III) and interactions of treatments with peer beliefs (models IV-VI).

|  |  | **I** |  | **II** |  | **III** |  |  | **IV** |  | **V** |  | **VI** |
| --- | --- | --- | --- | --- | --- | --- | --- | --- | --- | --- | --- | --- | --- |
| Scenario |  | 1 |  | 2 |  | 3 |  |  | 1 |  | 2 |  | 3 |
| P50 |  | 0.427 |  | -0.073 |  | 0.042 |  |  | 0.577 |  | -0.032 |  | -0.065 |
|  |  | (0.458) |  | (0.378) |  | (0.347) |  |  | (0.502) |  | (0.401) |  | (0.378) |
| P25 |  | -0.463 |  | -0.988*** |  | -1.030*** |  |  | -0.454 |  | -1.071*** |  | -1.071*** |
|  |  | (0.433) |  | (0.378) |  | (0.370) |  |  | (0.455) |  | (0.387) |  | (0.379) |
| P10 |  | 0.029 |  | -0.245 |  | -0.984*** |  |  | 0.174 |  | -0.273 |  | -0.891** |
|  |  | (0.430) |  | (0.372) |  | (0.373) |  |  | (0.461) |  | (0.383) |  | (0.380) |
| First-order beliefs |  | 0.227 |  | 0.210 |  | -0.435 |  |  | 0.188 |  | 0.161 |  | -0.369 |
|  |  | (0.317) |  | (0.295) |  | (0.285) |  |  | (0.341) |  | (0.305) |  | (0.297) |
| Second-order beliefs |  | 0.648* |  | 0.209 |  | 0.332 |  |  | 0.734* |  | 0.341 |  | 0.293 |
|  |  | (0.375) |  | (0.296) |  | (0.284) |  |  | (0.414) |  | (0.311) |  | (0.300) |
| Peer group deception |  | -1.037*** |  | -0.750*** |  | -0.678* |  |  | -1.135*** |  | -0.724*** |  | -0.771** |
|  |  | (0.311) |  | (0.269) |  | (0.355) |  |  | (0.346) |  | (0.274) |  | (0.373) |
| P50xPeer |  |  |  |  |  |  |  |  | 0.131 |  | -0.197 |  | -0.799 |
|  |  |  |  |  |  |  |  |  | (0.487) |  | (0.516) |  | (0.504) |
| P25xPeer |  |  |  |  |  |  |  |  | -0.005 |  | -0.117 |  | -0.601 |
|  |  |  |  |  |  |  |  |  | (0.424) |  | (0.44) |  | (0.509) |
| P10xPeer |  |  |  |  |  |  |  |  | -0.099 |  | -0.021 |  | -0.735 |
|  |  |  |  |  |  |  |  |  | (0.508) |  | (0.446) |  | (0.574) |
| Punishment considerations |  | 0.574* |  | -0.037 |  | 0.123 |  |  |  |  |  |  |  |
|  |  | (0.303) |  | (0.271) |  | (0.269) |  |  |  |  |  |  |  |
| Female |  | -0.049 |  | -0.291 |  | 0.214 |  |  |  |  |  |  |  |
|  |  | (0.314) |  | (0.273) |  | (0.265) |  |  |  |  |  |  |  |
| Age |  | -0.140** |  | -0.037 |  | -0.020 |  |  |  |  |  |  |  |
|  |  | (0.058) |  | (0.047) |  | (0.047) |  |  |  |  |  |  |  |
| Econ_bus |  | -0.235 |  | -0.329 |  | -0.134 |  |  |  |  |  |  |  |
|  |  | (0.314) |  | (0.276) |  | (0.276) |  |  |  |  |  |  |  |
| Funding |  | -0.280 |  | -0.074 |  | -0.449 |  |  |  |  |  |  |  |
|  |  | (0.332) |  | (0.281) |  | (0.278) |  |  |  |  |  |  |  |
| Siblings |  | -0.427 |  | 0.277 |  | -0.494 |  |  |  |  |  |  |  |
|  |  | (0.514) |  | (0.373) |  | (0.389) |  |  |  |  |  |  |  |
| Constant |  | 1.111*** |  | 0.842*** |  | 0.524** |  |  |  |  |  |  |  |
|  |  | (0.288) |  | (0.261) |  | (0.241) |  |  |  |  |  |  |  |
| Chi^2^ |  | 10.11 |  | 13.55 |  | 23.38 |  |  | 37.38 |  | 26.53 |  | 35.08 |
| Pseudo R^2^ |  | 0.08 |  | 0.09 |  | 0.14 |  |  | 0.28 |  | 0.17 |  | 0.21 |

Notes: *** *p*-value < 0.01; ** *p*-value < 0.05; * *p*-value < 0.1. Standard errors in parentheses. N=122.

Table C. Determinants of senders’ selection of honest messages including first-order beliefs (models I-III) and second-order beliefs (models IV-VI).

|  |  |  | **I** |  | **II** |  | **III** |  | **IV** |  | **V** |  | **VI** |
| --- | --- | --- | --- | --- | --- | --- | --- | --- | --- | --- | --- | --- | --- |
| Scenario |  |  | 1 |  | 2 |  | 3 |  | 1 |  | 2 |  | 3 |
| P50 |  |  | 0.116 |  | -0.048 |  | -0.224 |  | 0.274 |  | 0.046 |  | -0.202 |
|  |  |  | (0.448) |  | (0.385) |  | (0.361) |  | (0.457) |  | (0.390) |  | (0.368) |
| P25 |  |  | -1.002*** |  | -1.166*** |  | -1.287*** |  | -0.885** |  | -1.080*** |  | -1.250*** |
|  |  |  | (0.388) |  | (0.361) |  | (0.357) |  | (0.398) |  | (0.369) |  | (0.365) |
| P10 |  |  | -0.365 |  | -0.437 |  | -1.136*** |  | -0.340 |  | -0.402 |  | -1.060*** |
|  |  |  | (0.402) |  | (0.361) |  | (0.358) |  | (0.407) |  | (0.364) |  | (0.353) |
| First-order beliefs |  |  | 0.147 |  | 0.016 |  | -0.308 |  |  |  |  |  |  |
|  |  |  | (0.295) |  | (0.277) |  | (0.281) |  |  |  |  |  |  |
| Second-order beliefs |  |  |  |  |  |  |  |  | 0.606* |  | 0.435 |  | 0.164 |
|  |  |  |  |  |  |  |  |  | (0.351) |  | (0.294) |  | (0.285) |
| Female |  |  | -0.092 |  | -0.207 |  | 0.283 |  | -0.196 |  | -0.275 |  | 0.263 |
|  |  |  | (0.281) |  | (0.257) |  | (0.253) |  | (0.291) |  | (0.262) |  | (0.257) |
| Age |  |  | -0.113** |  | -0.050 |  | -0.003 |  | -0.120** |  | -0.051 |  | -0.001 |
|  |  |  | (0.050) |  | (0.045) |  | (0.045) |  | (0.051) |  | (0.045) |  | (0.044) |
| Econ_bus |  |  | -0.404 |  | -0.299 |  | -0.131 |  | -0.402 |  | -0.321 |  | -0.129 |
|  |  |  | (0.285) |  | (0.264) |  | (0.268) |  | (0.289) |  | (0.267) |  | (0.268) |
| Funding |  |  | 0.007 |  | 0.012 |  | -0.338 |  | 0.003 |  | -0.012 |  | -0.408 |
|  |  |  | (0.303) |  | (0.273) |  | (0.269) |  | (0.303) |  | (0.271) |  | (0.265) |
| Siblings |  |  | -0.179 |  | 0.223 |  | -0.564 |  | -0.018 |  | 0.315 |  | -0.551 |
|  |  |  | (0.452) |  | (0.357) |  | (0.382) |  | (0.458) |  | (0.362) |  | (0.387) |
| Constant |  |  | 3.844*** |  | 1.972* |  | 1.363 |  | 3.787*** |  | 1.798* |  | 1.044 |
|  |  |  | (1.249) |  | (1.087) |  | (1.082) |  | (1.245) |  | (1.052) |  | (1.056) |
| Chi^2^ |  |  | 18.55 |  | 17.03 |  | 29.67 |  | 21.49 |  | 19.26 |  | 28.8 |
| Pseudo R^2^ |  |  | 0.14 |  | 0.11 |  | 0.18 |  | 0.16 |  | 0.12 |  | 0.17 |

Notes: *** *p*-value < 0.01; ** *p*-value < 0.05; * *p*-value < 0.1. Standard errors in parentheses. N=122.

Table D. Determinants of senders’ selection of honest messages including peer beliefs (models I-III) and punishment considerations (models IV-VI).

|  |  |  | **I** |  | **II** |  | **III** |  | **IV** |  | **V** |  | **VI** |
| --- | --- | --- | --- | --- | --- | --- | --- | --- | --- | --- | --- | --- | --- |
| Scenario |  |  | 1 |  | 2 |  | 3 |  | 1 |  | 2 |  | 3 |
| P50 |  |  | 0.342 |  | -0.085 |  | -0.165 |  | 0.160 |  | -0.046 |  | -0.245 |
|  |  |  | (0.466) |  | (0.392) |  | (0.368) |  | (0.453) |  | (0.384) |  | (0.359) |
| P25 |  |  | -0.831** |  | -1.091*** |  | -1.188*** |  | -0.794* |  | -1.170*** |  | -1.262*** |
|  |  |  | (0.406) |  | (0.369) |  | (0.365) |  | (0.407) |  | (0.371) |  | (0.369) |
| P10 |  |  | -0.135 |  | -0.278 |  | -0.897** |  | -0.200 |  | -0.442 |  | -1.054*** |
|  |  |  | (0.426) |  | (0.375) |  | (0.365) |  | (0.413) |  | (0.366) |  | (0.360) |
| Peer group deception |  |  | -0.877*** |  | -0.713*** |  | -0.738** |  |  |  |  |  |  |
|  |  |  | (0.301) |  | (0.261) |  | (0.367) |  |  |  |  |  |  |
| Punishment considerations |  |  |  |  |  |  |  |  | 0.591** |  | -0.012 |  | 0.087 |
|  |  |  |  |  |  |  |  |  | (0.295) |  | (0.267) |  | (0.266) |
| Female |  |  | 0.023 |  | -0.225 |  | 0.254 |  | -0.033 |  | -0.210 |  | 0.299 |
|  |  |  | (0.288) |  | (0.263) |  | (0.255) |  | (0.287) |  | (0.259) |  | (0.253) |
| Age |  |  | -0.111** |  | -0.039 |  | -0.015 |  | -0.125** |  | -0.050 |  | -0.001 |
|  |  |  | (0.051) |  | (0.047) |  | (0.047) |  | (0.052) |  | (0.045) |  | (0.045) |
| Econ_bus |  |  | -0.259 |  | -0.300 |  | -0.133 |  | -0.358 |  | -0.301 |  | -0.115 |
|  |  |  | (0.298) |  | (0.27) |  | (0.271) |  | (0.291) |  | (0.267) |  | (0.269) |
| Funding |  |  | -0.128 |  | -0.036 |  | -0.486* |  | -0.022 |  | 0.015 |  | -0.403 |
|  |  |  | (0.312) |  | (0.277) |  | (0.272) |  | (0.304) |  | (0.269) |  | (0.265) |
| Siblings |  |  | -0.319 |  | 0.196 |  | -0.547 |  | -0.282 |  | 0.225 |  | -0.587 |
|  |  |  | (0.458) |  | (0.366) |  | (0.385) |  | (0.472) |  | (0.357) |  | (0.385) |
| Constant |  |  | 4.278*** |  | 2.132* |  | 1.976* |  | 3.844*** |  | 1.994* |  | 1.056 |
|  |  |  | (1.253) |  | (1.097) |  | (1.183) |  | (1.279) |  | (1.070) |  | (1.067) |
| Chi^2^ |  |  | 27.19 |  | 24.66 |  | 32.72 |  | 22.37 |  | 17.03 |  | 28.58 |
| Pseudo R^2^ |  |  | 0.21 |  | 0.16 |  | 0.19 |  | 0.17 |  | 0.11 |  | 0.17 |

Notes: *** *p*-value < 0.01; ** *p*-value < 0.05; * *p*-value < 0.1. Standard errors in parentheses. N=122.

Table E. Determinants of receivers’ acceptance of messages including further controls.

|  |  | **I** |  | **II** |  | **III** |
| --- | --- | --- | --- | --- | --- | --- |
| P50 |  | 0.252 |  | 0.544 |  | 0.299 |
|  |  | (0.345) |  | (0.538) |  | (0.616) |
| P25 |  | 0.000 |  | -0.122 |  | -0.544 |
|  |  | (0.340) |  | (0.603) |  | (0.762) |
| P10 |  | -0.184 |  | -0.443 |  | -1.072 |
|  |  | (0.335) |  | (0.557) |  | (0.668) |
| First-order beliefs |  |  |  | 2.983*** |  | 4.113*** |
|  |  |  |  | (0.583) |  | (0.999) |
| Relative payoff expectations |  |  |  | -1.134** |  | -1.779** |
|  |  |  |  | (0.509) |  | (0.744) |
| Peer group trust |  |  |  | 0.945** |  | 0.871* |
|  |  |  |  | (0.419) |  | (0.470) |
| Female |  |  |  |  |  | -1.053** |
|  |  |  |  |  |  | (0.535) |
| Age |  |  |  |  |  | -0.125* |
|  |  |  |  |  |  | (0.070) |
| Econ_bus |  |  |  |  |  | -0.029 |
|  |  |  |  |  |  | (0.498) |
| Funding |  |  |  |  |  | -0.365 |
|  |  |  |  |  |  | (0.556) |
| Siblings |  |  |  |  |  | 0.466 |
|  |  |  |  |  |  | (1.261) |
| Constant |  | 0.524** |  | - 0.796* |  | 2.398 |
|  |  | (0.241) |  | (0.463) |  | (1.962) |
| Chi^2^ |  | 1.67 |  | 95.19 |  | 102.27 |
| Pseudo R^2^ |  | 0.01 |  | 0.64 |  | 0.69 |

Notes: *** *p*-value < 0.01; ** *p*-value < 0.05; * *p*-value < 0.1. Standard errors in parentheses. N=122.
